# Chinese Herbal Medicine Usage Reduces Overall Mortality in HIV-Infected Patients With Osteoporosis or Fractures

**DOI:** 10.3389/fphar.2021.593434

**Published:** 2021-04-16

**Authors:** Mao-Wang Ho, Te-Mao Li, Ju-Pi Li, Jian-Shiun Chiou, Mu-Lin Chiu, Chao-Jung Chen, Chi-Fung Cheng, Fuu-Jen Tsai, Yang-Chang Wu, Ting-Hsu Lin, Chiu-Chu Liao, Shao-Mei Huang, Yu-Ning Lin, Chen-Hsing Chou, Wen-Miin Liang, Ying-Ju Lin

**Affiliations:** ^1^ Section of Infectious Diseases, Department of Internal Medicine, China Medical University Hospital, Taichung, Taiwan; ^2^ Department of Internal Medicine, School of Medicine, China Medical University, Taichung, Taiwan; ^3^ School of Chinese Medicine, China Medical University, Taichung, Taiwan; ^4^ School of Medicine, Chung Shan Medical University, Taichung, Taiwan; ^5^ Department of Pediatrics, Chung Shan Medical University Hospital, Taichung, Taiwan; ^6^ Department of Health Services Administration, China Medical University, Taichung, Taiwan; ^7^ Graduate Institute of Integrated Medicine, China Medical University, Taichung, Taiwan; ^8^ Proteomics Core Laboratory, Department of Medical Research, Genetic Center, China Medical University Hospital, Taichung, Taiwan; ^9^ Department of Biotechnology and Bioinformatics, Asia University, Taichung, Taiwan

**Keywords:** HIV, osteoporosis, fracture, overall mortality, Chinese herbal medicine, network analysis

## Abstract

The survival of patients with HIV has greatly improved, due to Anti-Retroviral Therapy (ART). However, long-term HIV survivors often develop serious bone abnormalities, possibly due to the interplay of osteoblasts, osteoclasts, HIV ad ART. We evaluated in a nation-wide study in Taiwan the effect of Chinese herbal medicine (CHM) on overall mortality in HIV patients with osteoporosis or fractures. Enrollment period was between 1998 and 2011. Patients with osteoporosis or fractures before the HIV infection, and those with less than 14 days CHM use, were excluded. This left 498 patients, 160 CHM users, 338 without CHM. Univariate Kaplan-Meier and multivariate Cox regression analysis were used to compare the overall mortality in these 2 groups. Due to the nature of Chinese medicine, CHMs inevitably varied. We therefore also used rule mining and network analysis to determine which major CHM clusters were prescribed to the patients. CHM users had a much Lower mortality (hazard ratio (HR) = 0.43, 95% confidence interval (CI): 0.24–0.77, *p* < 0.005) and higher survival (*p* = 0.004, log-rank test). Although the CHMs greatly varied, network analysis identified one main cluster of strongly related CHM combinations (Chuan-Xiong-Cha-Tiao-San (CXCTS), Gan-Cao (GC; *Glycyrrhiza uralensis Fisch*.), Liu-He-Tang (LHT), Huang-Qin-Tang (HQT), Jia-Wei-Ping-Wei-San (JWPWS), and Dang-Gui-Long-Hui-Wan (DGLHuiW)). CHM as an additional treatment strongly improves overall survival in HIV-infected patients with osteoporosis and fractures.

## Introduction

With antiretroviral therapy (ART), HIV-positive and negative patients have similar lifespans (Pham and Mesplede, 2018; Zhang et al., 2019). Patients with HIV/AIDS who receive ART demonstrate delayed AIDS progression, improved quality of life, and lower all-cause mortality (Antiretroviral Therapy Cohort Collaboration, 2017; Lu et al., 2018). ART suppresses viral replication; it does not eliminate the virus. Discontinuation of ART results in drug resistance of HIV, viral reactivation, and disease progression (Meintjes et al., 2017; Dubrocq and Rakhmanina, 2018). Long-term living with HIV and ART use in HIV-infected patients are associated with adverse effects. These adverse effects include hyperlipidemia, cardiovascular disease, bone related abnormalities, diabetes, and renal disease (Kwong et al., 2006; De Wit et al., 2008; Capeau et al., 2012; Achhra et al., 2016; Grant et al., 2016; Hoy and Young, 2016; Ahmad et al., 2017; Dorjee et al., 2017; Hoy et al., 2017; Tsai et al., 2017; Nan et al., 2018).

Bone related abnormalities including low bone density, osteomalacia, osteonecrosis, osteopenia, osteoporosis, and fracture (Hoy and Young, 2016; Ahmad et al., 2017). Osteoporosis is a multifactorial systemic skeletal disease with low bone density, degeneration of bone architecture, bone fragility, and consequent increased risk of fracture (Ji and Yu, 2015; Sozen et al., 2017). A number of studies report that lower bone density was observed in HIV-infected patients when compared with non-infected individuals (Bruera et al., 2003; Amiel et al., 2004; Arnsten et al., 2007). The pathological mechanism between HIV and/or ART and bone related abnormalities remain to be elucidated, but are probably due to HIV and ART affecting the interactions between osteoclasts and osteoblasts. Furthermore, the loss of bone mineral density is frequently observed in HIV-infected patients with ART (Duvivier et al., 2009; van Vonderen et al., 2009; Grant et al., 2016; Hoy et al., 2017; Chisati et al., 2020b). HIV-infected patients placed on protease inhibitor (PI) regimens demonstrate bone loss in the spine, while the nucleoside/nucleotide reverse transcriptase inhibitor (NRTI) regimen is associated with bone loss at the hip (Hoy et al., 2017).

Chinese herbal medicines (CHMs) are often used to treat bone related diseases, as they show anti-inflammatory, anti-osteopenia, anti-osteoporotic, and promote fracture healing activities (Chow et al., 1982; Chen et al., 2005; Li et al., 2011; Ma et al., 2011; Xiang et al., 2011; Shih et al., 2012; Wong et al., 2013; He and Shen, 2014; Mukwaya et al., 2014; Lin et al., 2015; Zhang et al., 2016; Hsiao et al., 2017; Wang et al., 2018b; Xi et al., 2018; Cheng et al., 2019a; Cheng et al., 2019b). However, none of these studies have been carried out in prospective randomized clinical trials in humans. These results encourage to analyze if CHM as additional therapy to improve osteoporosis and fractures management and survival among HIV-infected patients. We therefore analyzed in a population-based nationwide database from Taiwan, what the effect was of CHM treatment-or-not on the overall mortality in HIV-infected patients with osteoporosis or fractures.

## Materials and Methods

### Study Participants

**FIGURE 1:**
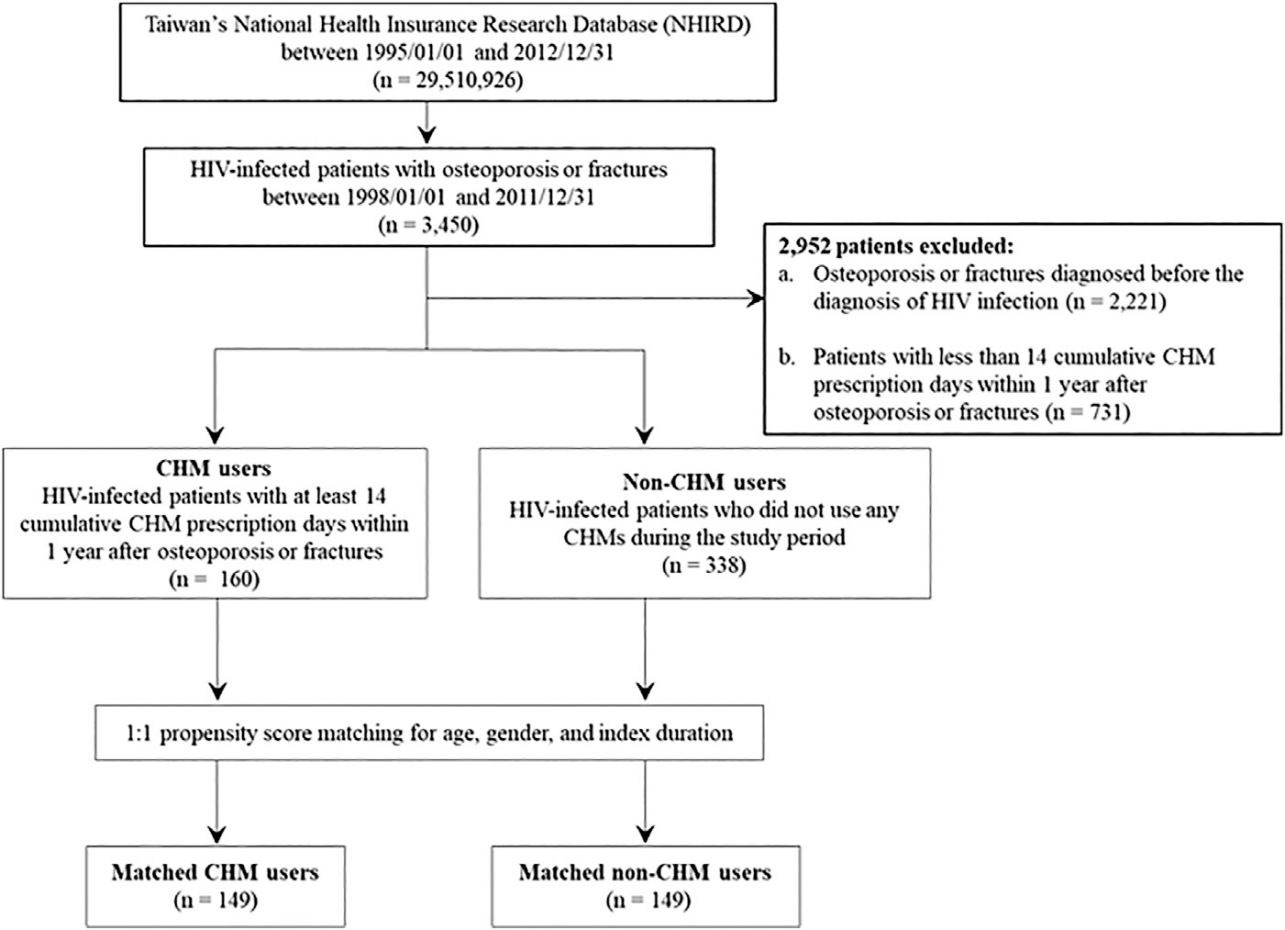
Flowchart for the enrollment of CHM and non-CHM users in HIV-infected patients with osteoporosis or fractures. CHM, Chinese herbal medicine.

**FIGURE 2:**
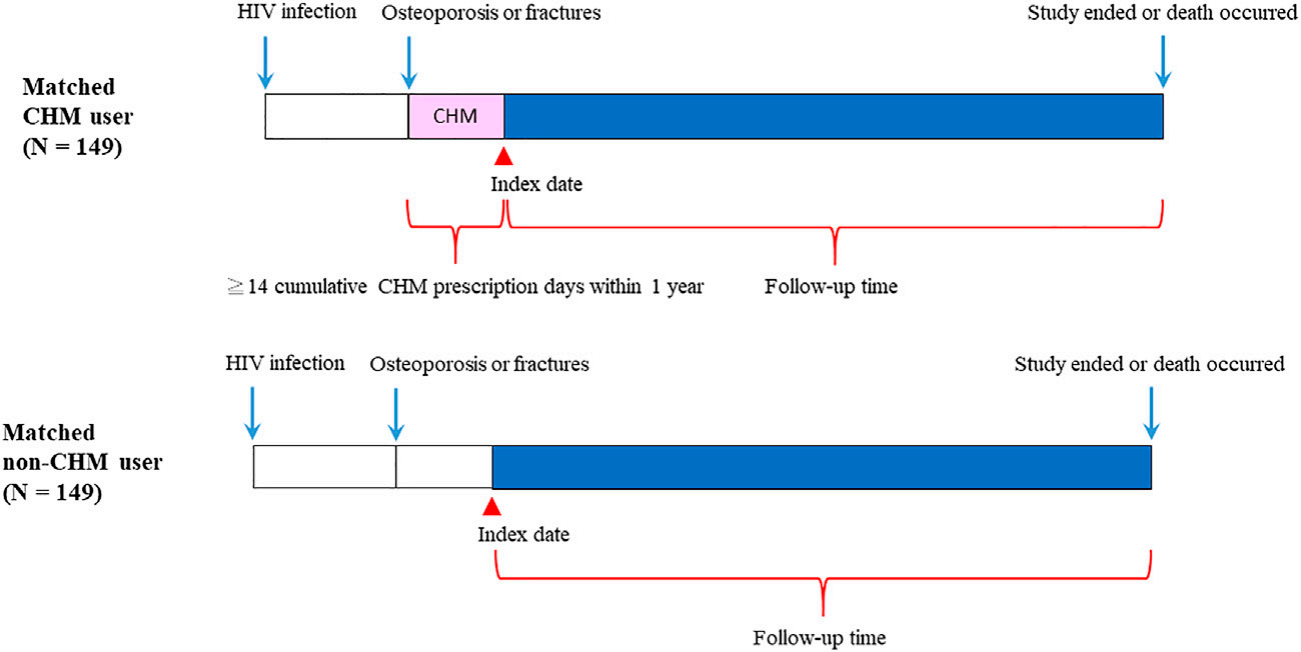
Follow-up times of CHM and non-CHM users in HIV-infected patients with osteoporosis or fractures. CHM, Chinese herbal medicine.

This is a longitudinal study spanning 1995 through 2012 using the database of National Health Insurance Research Database in Taiwan (NHIRD; http://nhird.nhri.org.tw/). From the database, 3450 anonymized HIV-infected patients with osteoporosis or fractures were further identified during the period between 1998 and 2011 (Figures 1,2) (the International Classification of Disease, 9^th^ Revision, Clinical Modification (ICD-9-CM) codes for HIV infection: 042-044, V08; ICD-9-CM codes for osteoporosis: 7330; ICD-9-CM codes for fractures: 8050, 8052, 8054, 8056, 8058, 8060, 8062, 8064, 8068, 8070, 8072, 8074, 8075, 8080, 8082, 8084, 8088, 8090, 8100, 8110, 8120, 8122, 8124, 8130, 8132, 8134, 8138, 8140, 8200, 8202, 8208, 8210, 8212, 8220, 8230, 8232, 8238, 8240, 8242, 8244, 8246, and 8248). Of these, 2952 excluded for: 1) osteoporosis or fractures diagnosed before the diagnosis of HIV infection (*n* = 2,221); 2) less than 14 CHM cumulative prescription days within 1 year after osteoporosis or fractures (*n* = 731).

The flowchart for the selection for HIV-infected patients with osteoporosis or fractures is shown in Figures 1,2. Patients were initially diagnosed with HIV infection followed by osteoporosis or fractures (Figure 2). Between January 01, 1998 and December 31, 2011, 3450 HIV-infected patients with osteoporosis or fractures were identified (Figure 1). After exclusion, there were 498 patients with osteoporosis or fractures, including 160 CHM and 338 non-CHM users (Figure 1). The CHM users were defined as the patients who received CHMs for at least 14 days in the 12 months after osteoporosis or fractures (Figures 1,2).

Patients were classified as CHM users when they received more than 14 CHM cumulative prescription days among the first year after osteoporosis or fractures (*n* = 160, Figures 1,2). The index date started after the 14 cumulative CHM prescription days were accomplished (Figure 2). The CHM users received CHM therapies during the study period (Supplementary Table S2). On the other hand, the controls were classified as non-CHM users when they did not receive any CHMs for the study period (*n* = 338). To reduce potential confounding factors, these two groups were matched for age, gender, and index duration using the propensity score matching method (1:1 ratio) (Table 1). This resulted in 149 CHM and 149 non-CHM users (Figures 1,2; Table 1).

**TABLE 1:** Demographic characteristics of HIV-infected patients with osteoporosis or fractures according to Chinese herbal medicine usage in Taiwan.

Characteristics	Total subjects		*p*-value	Matched subjects		*p*-value
	CHM users (N = 160)	Non-CHM users (N = 338)		CHM users (N = 149)	Non-CHM users (N = 149)	
	N (%)	N (%)		N (%)	N (%)	
Age (years old; Mean ± SD)	44.81 ± 15.2	39.53 ± 13.44	**<0.001**	44.3 ± 15.06	42.62 ± 15.17	0.339
0≦ Age <30	24 (15.00%)	60 (17.75%)	**0.007**	24 (16.11%)	27 (18.12%)	0.308
30≦ Age <40	45 (28.13%)	136 (40.24%)		41 (27.52%)	51 (34.23%)	
40≦ Age	91 (56.88%)	142 (42.01%)		84 (56.38%)	71 (47.65%)	
Gender			**<0.001**			0.903
Male	105 (65.63%)	280 (82.84%)		99 (66.44%)	98 (65.77%)	
Female	55 (34.38%)	58 (17.16%)		50 (33.56%)	51 (34.23%)	
Index duration (day; Mean ± SD)	1307.02 ± 1046.84	1087.01 ± 923.88	**0.018**	1283.97 ± 1041.17	1321.81 ± 1131.97	0.764
ART usage			0.057			0.310
Non-ART use	154 (96.25%)	334 (98.82%)		143 (95.97%)	146 (97.99%)	
ART use	6 (3.75%)	4 (1.18%)		6 (4.03%)	3 (2.01%)	
Charlson comorbidity index (CCI)			0.331			0.931
0	38 (23.75%)	78 (23.08%)		37 (24.83%)	36 (24.16%)	
1	92 (57.5%)	213 (63.02%)		85 (57.05%)	88 (59.06%)	
≥2	30 (18.75%)	47 (13.91%)		27 (18.12%)	25 (16.78%)	
Comorbidities						
Myocardial infarction	1 (0.63%)	1 (0.3%)	0.588	1 (0.67%)	0 (0.00%)	0.316
Congestive heart failure	5 (3.13%)	4 (1.18%)	0.129	4 (2.68%)	4 (2.68%)	1.000
Peripheral vascular disease	4 (2.5%)	5 (1.48%)	0.425	4 (2.68%)	4 (2.68%)	1.000
Cerebrovascular disease	12 (7.5%)	10 (2.96%)	**0.021**	9 (6.04%)	5 (3.36%)	0.273
Dementia	2 (1.25%)	2 (0.59%)	0.442	1 (0.67%)	2 (1.34%)	0.562
Chronic pulmonary disease	34 (21.25%)	33 (9.76%)	**<0.001**	30 (20.13%)	22 (14.77%)	0.222
Rheumatic disease	4 (2.5%)	4 (1.18%)	0.275	3 (2.01%)	3 (2.01%)	1.000
Peptic ulcer disease	38 (23.75%)	43 (12.72%)	**0.002**	36 (24.16%)	24 (16.11%)	0.083
Mild liver disease	41 (25.63%)	79 (23.37%)	0.583	38 (25.5%)	34 (22.82%)	0.588
Diabetes without chronic complication	19 (11.88%)	20 (5.92%)	**0.021**	17 (11.41%)	9 (6.04%)	0.101
Diabetes with chronic complication	4 (2.5%)	6 (1.78%)	0.590	4 (2.68%)	4 (2.68%)	1.000
Renal disease	6 (3.75%)	2 (0.59%)	**0.009**	6 (4.03%)	1 (0.67%)	0.056
Moderate or severe liver disease	2 (1.25%)	0 (0.00%)	**0.039**	2 (1.34%)	0 (0.00%)	0.156

ART, antiretroviral therapies; CCI, Charlson comorbidity index; CHM, Chinese herbal medicine; N, number; SD, standard deviation.

*p*-values were obtained by the chi-square test; *p*-values for age and duration were obtained by the un-paired Student t test.

Significant *p*-values (*p* < 0.05) are highlighted in bold italic font.

Index duration was from the diagnosed date of HIV infection to the diagnosed date of osteoporosis or fractures (day; Mean ± SD).

Comorbidities present in the patients prior to their subsequent HIV diagnosis were defined as follows: myocardial infarction (ICD-9-CM:410.x, 412*), congestive heart failure (ICD-9-CM: 428.x), peripheral vascular disease (ICD-9-CM: 441.x*, 443.9*, 785.4*, V43.4*, 38.48(P)), cerebrovascular disease (ICD-9-CM: 430.x-438.x*), dementia (ICD-9-CM: 290.x*), chronic pulmonary disease (ICD-9-CM: 490.x-496.x*, 500.x-505.x*, 506.4*), rheumatic disease (ICD-9-CM: 710.0, 710.1*, 710.4*, 714.0–714.2*, 714.81, 725.x*), peptic ulcer disease (ICD-9-CM: 531.x-534.x), mild liver disease (ICD-9-CM: 571.2*, 571.4–571.6*), diabetes without chronic complication (ICD-9-CM: 250.0–250.3*, 250.7*), diabetes with chronic complication (ICD-9-CM: 250.4–250.6*), renal disease (ICD-9-CM: 582.x*, 583–583.7*, 585.x*, 586.x*, 588.x*), and moderate or severe liver disease (ICD-9-CM: 456.0–456.21*, 572.2–572.8*).

Among these patients, the characteristics included age, gender, index duration (from the HIV infection diagnosed date to the diagnosed date of osteoporosis or fractures), Charlson comorbidity index (CCI), and comorbidities (Table 1). In this study, comorbidities were defined before HIV infection (Table 1). The ART usage was defined before the diagnosed date of osteoporosis or fractures (Table 1). The study was approved by the Institutional Review Board of the China Medical University Hospital (The ethics approval number: CMUH107-REC3-074(CR1)).

### Chinese Herbal Medicine, Association Rule Mining, and Network Analysis

These CHMs are prescribed by licensed and experienced traditional Chinese medicine doctors in Taiwan, and they are served as traditional Chinese medicine in health care systems in Taiwan. CHMs include single herbs and herbal formulae. A single herb is made from the flower, root, stem, or leaf of a given plant. It is also made from an organ of an animal, insect, or mineral source. The herbal formulae are mixtures of a minimum of two single herbs. The CHM composition, frequency, and usage patterns are shown in Supplementary Table S1. CHMs are produced by pharmaceutical Good Manufacturing Practice companies with in Taiwan.

Association rule mining was performed, as previously described, using SAS software (version 9.4; SAS Institute, Cary, NC, United States). This association rule mining has been applied to discover studies in the relationships of these CHM prescriptions (Chen et al., 2014; Cheng et al., 2019b; Tsai et al., 2019). Chinese herbal medicine (CHM) product X (CHM_X) and CHM product Y (CHM_Y) were shown as the “items,” respectively. The CHM prescriptions were used as the “transactions,” with co-occurrences of CHM_X and CHM_Y (Table 3). This expression shows the relationship between the occurrences of CHM_X and CHM_Y. The strength of the association using this technique was expressed as support, confidence, and lift. Support is a measure of whether an association between CHM_X and CHM_Y happened by chance. The support (X) (%) value is the calculated joint probability of having both of CHM_X and CHM_Y, which is (the frequency of CHM_X and CHM_Y/total number of prescription) × 100%. Confidence is an indicator of how often CHM_Y appeared in transactions that contained CHM_X. The confidence value (CHM_X → CHM_Y; %) is the calculated conditional probability of having a prescription of CHM_Y among those who already have the prescription of CHM_X, which is given as (frequency of CHM_X and CHM_Y/frequency of CHM_X) x 100%. Lift is the ratio of observed support to expected support when X and Y are independent. The lift value is the confidence (CHM_X → CHM_Y) (%)/P (Y) (%) or confidence (CHM_Y → CHM_X) (%)/P (X) (%). A lift value greater than 1 indicates that the occurrences between the two CHM products are dependent and suggests a strong co-occurrence relationship between CHM_X and CHM_Y.

**FIGURE 3:**
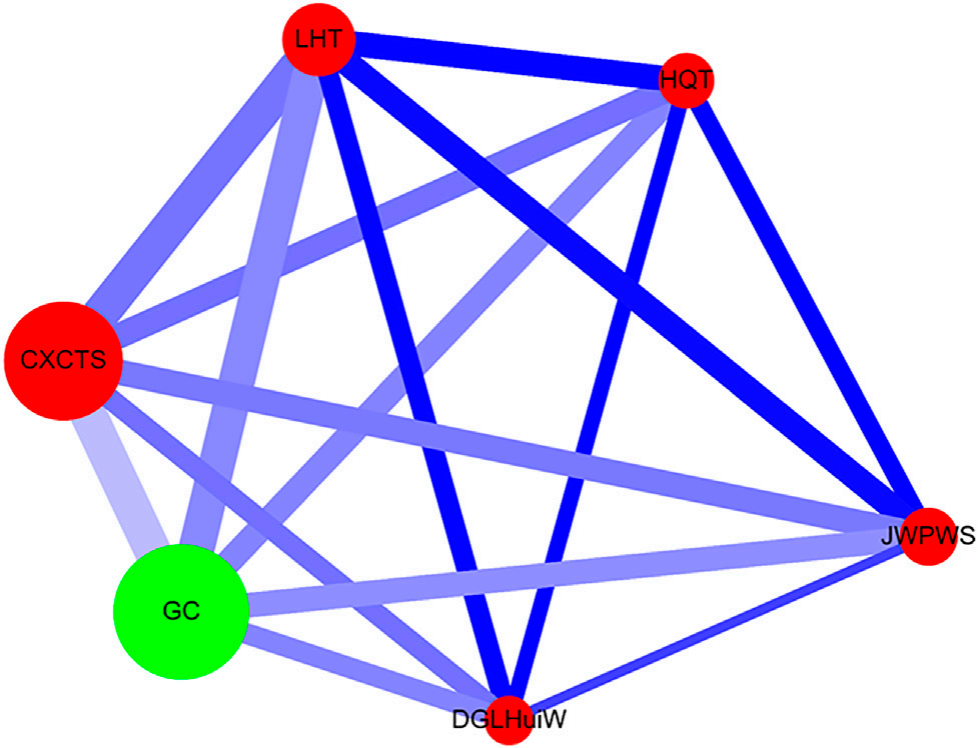
Network analysis for CHM prescription pattern in HIV-infected patients with osteoporosis or fractures. The lines connecting CHMs represent the support value: thicker lines represent higher support values (Support (X) (%)). The line color between CHMs shows the lift value: darker lines represent stronger connections with higher lift values. The red circle represents herbal formulas, while the green circle represents single herbs. The size of the circle for each CHM shows its prescription frequency: larger circles indicate higher prescription frequency. Support (X) (%) = Frequency of prescriptions of X and Y products/total prescriptions x 100%. Lift = Confidence (X → Y) (%)/P (Y) (%). Confidence (X → Y) (%) = Frequency of prescriptions of X and Y products/Frequency of prescriptions of X product x 100%. P (Y) (%) = Frequency of prescriptions of Y product/total prescriptions x 100%. CHM, Chinese herbal medicine; CXCTS, Chuan-Xiong-Cha-Tiao-San; DGLHuiW, Dang-Gui-Long-Hui-Wan; GC, Gan-Cao; HQT, Huang-Qin-Tang; JWPWS, Jia-Wei-Ping-Wei-San; LHT, Liu-He-Tang.

Network analysis was performed as previously described (Cheng et al., 2019a; Cheng et al., 2019b) (Figure 3). The single herb is expressed as a green circle, and the herbal formula is shown as a red circle. The prescription frequency of the single herb or herbal formula is shown (Supplementary Table S2) and is denoted as the circle size. The support value (%) (between CHM_X and CHM_Y) is shown in Table 3 and is expressed as the line size. The lift value is also shown in Table 3 and is represented as the line color. The connection strength between the paired CHM products is shown as the line size and line color. All data were employed using Cytoscape software (https://cytoscape.org/, version 3.7.0).

### Statistical Analysis

**FIGURE 4:**
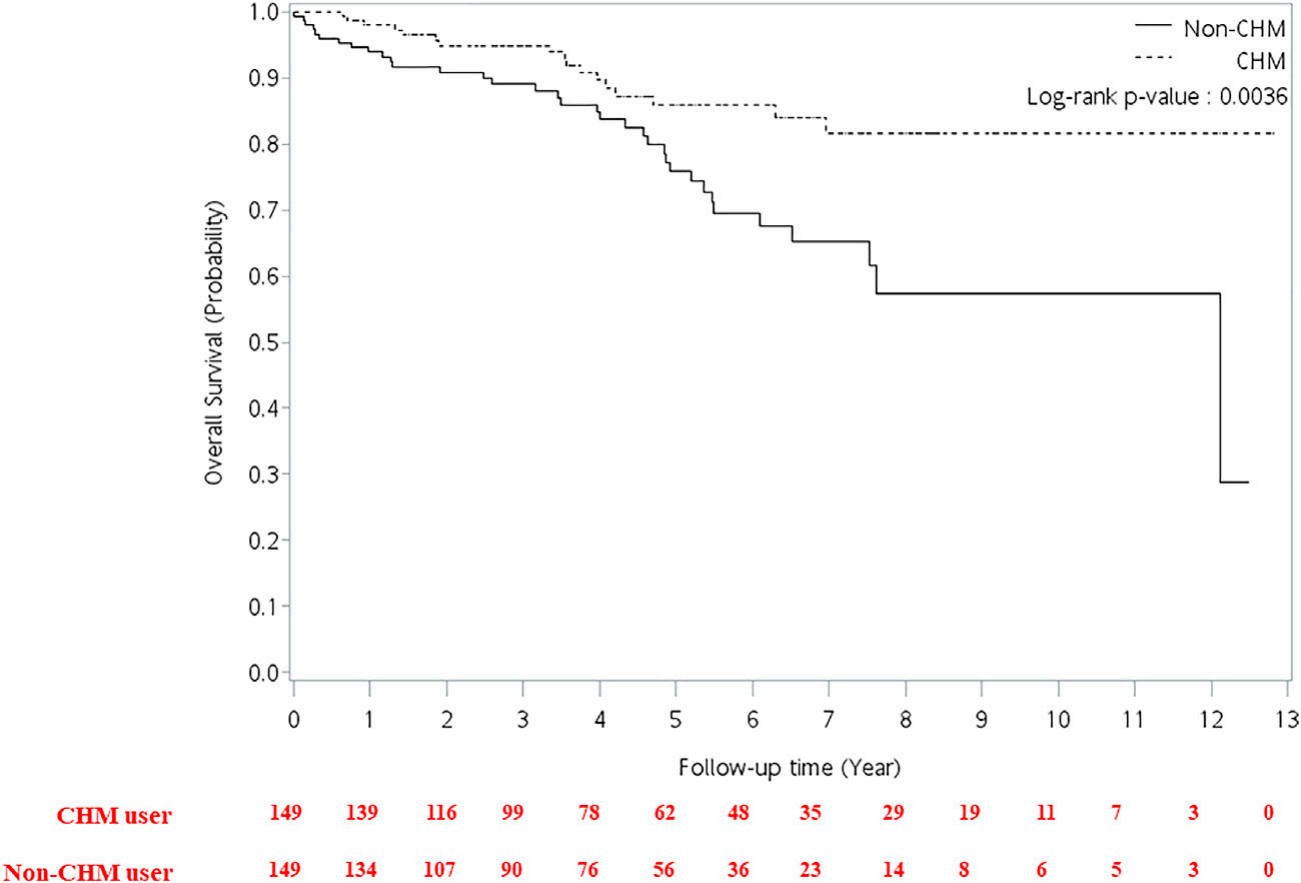
Cumulative incidence of overall survival between CHM and non-CHM users in HIV-infected patients with osteoporosis or fractures. CHM, Chinese herbal medicine.

Age was expressed as continuous data (years, mean ± SD) and categorical data (numbers (percentages)) (Table 1). Index duration was expressed as continuous data (from the diagnosed date of HIV infection to the diagnosed date of osteoporosis or fractures) (day, Mean ± SD) (Table 1). Gender, antiretroviral therapies (ART) usage, Charlson comorbidity index (CCI) and comorbid conditions were expressed as categorical data (numbers (percentages)) (Table 1). The un-paired Student t-test was applied in continuous data (Supplementary Table S2). The Chi-squared test was used in categorical data. Univariate (crude) and multivariate (adjusted) Cox proportional hazard models were employed to evaluate the risk of overall mortality (Table 2). Multivariate-adjustments include age, gender, CHM use, ART use, and CCI (Table 2). For survival analysis, Kaplan-Meier method and the log-rank test were performed (Figure 4; Supplementary Table S4, S5). All data and statistical analyses were employed using SAS software (version 9.4; SAS Institute, Cary, NC, United States).

**TABLE 2:** Cox proportional hazard models for overall mortality in HIV-infected patients with osteoporosis or fractures.

Variable	Crude			Adjusted		
	HR	95% CI	*p*-value	aHR	95% CI	*p*-value
Age, per year	1.02	(1.00–1.04)	**0.0299**	1.02	(1.00–1.04)	**0.0173**
Female (vs. Male)	0.51	(0.26–0.99)	**0.0465**	0.79	(0.44–1.42)	0.4279
CHM use (vs. Non-CHM use)	0.39	(0.21–0.70)	**0.0018**	0.43	(0.24–0.77)	**0.0047**
ART use (vs. Non-ART use)	1.21	(0.36–4.14)	0.7582	1.25	(0.38–4.09)	0.7087
Charlson comorbidity index (CCI)_1 (vs. 0)	1.32	(0.61–2.88)	0.4777	1.39	(0.65–2.99)	0.3982
Charlson comorbidity index (CCI)_≥2 (vs. 0)	2.95	(1.26–6.88)	**0.0125**	3.79	(1.69–8.51)	**0.0012**

ART, antiretroviral therapies; aHR, adjusted hazard ratio; CI, confidence interval; CCI, Charlson comorbidity index; CHM, Chinese herbal medicine; HR, hazard ratio.

*p*-*value* (*p* < 0.05) was shown in bold italic font.

Adjusted factors: age, gender, CHM use, and Charlson comorbidity index.

## Results

### Basic Characteristics

The 160 CHM users received CHM therapies during the study period (Supplementary Table S3). The other 338 patients did not use any CHM at all. The demographic characteristics of total subjects are shown in Table 1. When compared with the 338 non-CHM users, the 160 CHM users were slightly older, more often females, had a longer index duration between HIV diagnosis date and the osteoporosis or fractures date, and had more often comorbidities (*p* < 0.05). To prevent the effects of these confounding factors, propensity score matching (1:1 ratio) was applied to match the two groups for age, gender, and index duration. After matching, each group had 149 HIV-infected patients with osteoporosis or fractures (Figures 1,2; Table 1).

### Risk of Overall Mortality

The risk of overall mortality in patients with osteoporosis or fractures was evaluated by Cox proportional hazard models (Table 2). For univariate (crude) Cox proportional hazard model, there were differences in age, gender, CHM use, and comorbidities (*p* < 0.05). The univariate (crude) Cox proportional hazard model showed that patients showed a higher risk of overall mortality per year increase in age (Table 2; hazard ratio (HR): 1.021, 95% confidence interval (CI): 1.00–1.04, *p* = 0.0299). Female patients had a lower risk of overall mortality than male patients (HR: 0.51, 95% CI: 0.26–0.99, *p* = 0.0465). The CHM users had a lower risk of overall mortality than non-CHM users (HR: 0.39, 95% CI: 0.21–0.70, *p* = 0.0018). Patients with Charlson comorbidity index (CCI) ≥ 2 showed a higher risk of overall mortality than those who did not have any comorbidities (HR: 2.95, 95% CI: 1.26–6.88, *p* = 0.0125).

The multivariate Cox proportional hazard model showed that patients had a higher risk of overall mortality per year increase in age after adjusting for gender, CHM use, and Charlson comorbidity index (Table 2; adjusted hazard ratio (aHR): 1.02, 95% CI: 1.00–1.04, *p* = 0.0173). The CHM users had a lower risk of overall mortality than non-CHM users after adjusting for age, gender, and Charlson comorbidity index (aHR: 0.43, 95% CI: 0.24–0.77, *p* = 0.0047). Patients with Charlson comorbidity index (CCI) ≥ 2 showed a higher risk of overall mortality than those who did not have any comorbidities after adjusting for age, gender, and CHM use (aHR: 3.79, 95% CI: 1.69–8.51, *p* = 0.0012). Kaplan-Meier survival plots exhibited that there was a difference in the cumulative incidences of overall survival between the CHM and non-users (Figure 4; *p* = 0.0036, log-rank test). The cumulative incidence of overall survival was significantly higher in CHM users.

### CHM Prescription Pattern and Network Analysis

The commonly prescribed CHM products and compositions are listed for the HIV-infected patients with osteoporosis or fractures in Supplementary Table S1. LC-MS/MS analysis of active component standards and these 6 herbal extracts are also shown in Supplementary Figures S1–S6. According to the frequency of prescriptions (Supplementary Table S1), Chuan-Xiong-Cha-Tiao-San (CXCTS) was the most commonly herbal formula. The second and third formulas were Liu-He-Tang (LHT) and Jia-Wei-Ping-Wei-San (JWPWS), respectively. Gan-Cao (GC; *Glycyrrhiza uralensis Fisch.*) was the most commonly single herb.

Association rule analysis showed the 10 most commonly co-prescriptions of CHM products for HIV-infected patients with osteoporosis or fractures (Table 3). Higher levels of support, confidence, and lift values suggested stronger associations between the paired CHM products. According to the frequency of prescriptions, support, confidence, and lift values (Table 3), the most commonly used paired CHM products were Chuan-Xiong-Cha-Tiao-San (CXCTS) → Gan-Cao (GC; *Glycyrrhiza uralensis Fisch.*) (first co-prescription frequency: 172, support: 4.59%, confidence: 55.31%, lift: 5.75), followed by Liu-He-Tang (LHT) → CXCTS (second co-prescription frequency: 171, support: 4.57%, confidence: 97.71%, lift: 11.77), and LHT → GC (third co-prescription frequency: 169, support: 4.51%, confidence: 96.57%, lift: 10.05).

**TABLE 3:** Ten most commonly used co-prescriptions of CHM products for HIV-infected patients with osteoporosis or fractures in Taiwan.

CHM products (LHS, X)	Chinese name	Frequency of prescriptions of X product	Dosage of X product		CHM products (RHS, Y)	Chinese name	Frequency of prescriptions of Y product	Dosage of Y product	Frequency of prescriptions of X and Y products	Support (X) (%)	Confidence (X → Y) (%)	Lift
Chuan-Xiong-Cha-Tiao-San (CXCTS)	川芎茶調散	311	12,032.59	→	Gan-Cao (GC)	甘草	360	2556	172	4.59	55.31	5.75
Liu-He-Tang (LHT)	六和湯	175	6020	→	Chuan-Xiong-Cha-Tiao-san (CXCTS)	川芎茶調散	311	12,032.59	171	4.57	97.71	11.77
Liu-He-Tang (LHT)	六和湯	175	6020	→	Gan-Cao (GC)	甘草	360	2556	169	4.51	96.57	10.05
Huang-Qin-Tang (HQT)	黃芩湯	124	3162	→	Chuan-Xiong-Cha-Tiao-San (CXCTS)	川芎茶調散	311	12,032.59	124	3.31	100.00	12.04
Huang-Qin-Tang (HQT)	黃芩湯	124	3162	→	Gan-Cao (GC)	甘草	360	2556	124	3.31	100.00	10.40
Jia-Wei-Ping-Wei-San (JWPWS)	加味平胃散	131	1591.65	→	Liu-He-Tang (LHT)	六和湯	175	6020	124	3.31	94.66	20.26
Jia-Wei-Ping-Wei-San (JWPWS)	加味平胃散	131	1591.65	→	Chuan-Xiong-Cha-Tiao-San (CXCTS)	川芎茶調散	311	12,032.59	124	3.31	94.66	11.40
Huang-Qin-Tang (HQT)	黃芩湯	124	3162	→	Liu-He-Tang (LHT)	六和湯	175	6020	122	3.26	98.39	21.05
Jia-Wei-Ping-Wei-San (JWPWS)	加味平胃散	131	1591.65	→	Gan-Cao (GC)	甘草	360	2556	121	3.23	92.37	9.61
Dang-Gui-Long-Hui-Wan (DGLHuiW)	當歸龍薈丸	105	1755.6	→	Chuan-Xiong-Cha-Tiao-San (CXCTS)	川芎茶調散	311	12,032.59	105	2.80	100.00	12.04

CHM, Chinese herbal medicine; LHS, left-hand-side; RHS, right-hand-side.

Total prescriptions = 3745.

Dosage of X or Y products = Average drug dose per day (g) × Average duration for prescription (days) × Frequency of prescriptions (Supplementary Table S1).

Support (X) (%) = Frequency of prescriptions of X and Y products/total prescriptions × 100%.

Confidence (X → Y) (%) = Frequency of prescriptions of X and Y products/Frequency of prescriptions of X product × 100%.

P (Y) (%) = Frequency of prescriptions of Y product/total prescriptions × 100%.

Lift = Confidence (X → Y) (%)/P (Y) (%).

Network analysis showed the CHM prescription network for patients with osteoporosis or fractures (Figure 3; Supplementary Figure S7). There were 149 patients who used 3,745 prescriptions by traditional Chinese medicine doctors (Table 3). Network analysis showed one main CHM cluster, including CXCTS, GC, LHT, HQT, JWPWS, and DGLHuiW. Our results show that these 6 CHMs are important for HIV-infected patients with osteoporosis or fractures.

## Discussion

Long-term living with HIV and ART use in HIV-infected patients are associated with adverse effects including bone related abnormalities. In this study, we investigated the effect of CHMs on the overall mortality in HIV-infected patients with osteoporosis or fractures in Taiwan. We found that CHM usage reduced the overall mortality for these patients. We also described their CHM prescription network; these included CXCTS, GC, LHT, HQT, JWPWS, and DGLHuiW. CHM treatment exhibited lower risks of overall mortality for HIV-infected patients with osteoporosis or fractures in Taiwan.

Reduced bone mineral density is observed in HIV-infected patients on ART therapy (Duvivier et al., 2009; van Vonderen et al., 2009; Grant et al., 2016; Hoy et al., 2017; Chisati et al., 2020b). Furthermore, Chisati et al., reported that low bone mineral density was also associated with low levels of physical activity among these patients (Chisati et al., 2020b). Maximal strength training for physical activity improves bone mineral density for people living with HIV and receiving ART (Chisati et al., 2020a). In this study, we observed that among HIV-infected patients with osteoporosis or fractures, CHM users showed a lower risk of overall mortality after adjusting for age, gender, ART use, and CCI. The cumulative incidence of overall survival was higher in CHM users, especially different between 4 and 8 years. These CHM users received CHM therapies during the study period (Supplementary Table S3); the non-CHM users did not receive any CHMs for the study period. CHMs may exhibit bone protection effect after long-term treatment. Studies have also suggested that CHM may be beneficial for bone metabolism through osteopenia prevention, anti-osteoporotic activities, promotion of fracture healing, and inhibition of inflammation (Chow et al., 1982; Chen et al., 2005; Li et al., 2011; Ma et al., 2011; Xiang et al., 2011; Wong et al., 2013; He and Shen, 2014; Zhang et al., 2016; Hsiao et al., 2017; Wang et al., 2018b; Xi et al., 2018; Lee et al., 2019). Among these studies, there were two review studies reported in human beings. Zhang et al., reported that there were 33 Traditional Chinese medicine (TCM) formulas commonly used to treat osteoporosis, exhibiting anti-osteoporotic effects in humans and animals (Zhang et al., 2016). Wang et al., reported that a natural compound from the traditional Chinese medicinal herb was effective in preventing postmenopausal osteoporosis observed from a 24-months randomized double-blind placebo-controlled clinical trial in humans (Wang et al., 2018b). Our study identified one main CHM cluster, which includes CXCTS, GC, LHT, HQT, JWPWS, and DGLHuiW.

Therapeutic approaches for treating osteoporosis or fracture inhibit further loss of bone density and strength (Kling et al., 2014). The bisphosphonates (Lewiecki, 2010), which are anti-bone resorption medications, are among the major clinical pharmacological treatments. Bisphosphonates, including alendronate, risedronate, ibandronate, and zoledronate have strong affinities for hydroxyapatite in bone and a long skeletal half-life; therefore, they inhibit bone resorption (Pazianas et al., 2014). Inhibition of osteoclast differentiation resulting in the suppression of bone resorption is one of the potential therapy targets for anti-osteoporotic and anti-fracture activities (Weivoda et al., 2020). Chinese herbs and their related natural compounds may prevent osteoporosis or fractures via inhibition of osteoclast activities. Ferulic acid is one of the natural compounds of Chuan-Xiong (CX; *Rhizoma Chuanxiong*; *Ligusticum sinense Oliv.*) (Lv et al., 2010; Li et al., 2012), and it is a component of CXCTS, Zhi-Ban-Xia (ZBX; *Rhizoma Pinelliae Preparatum*; *Pinellia ternata (Thunb.) Makino*) (Han et al., 2007), LHT, Huang-Qin (HQin; *Radix Scutellariae*; *Scutellaria baicalensis Georg*i) (Lu et al., 2011), HQT, Dang-Gui (DG; *Radix Angelicae Sinensi*; *Angelica sinensis (Oliv.) Diels*) (Giacomelli et al., 2017), and DGLHuiW. Ferulic inhibits osteoclast differentiation (Sagar et al., 2016; Doss et al., 2018) and the RANKL dependent NF-kB signaling pathway (Doss et al., 2018). Gallic acid (also known as 3,4,5-trihydroxybenzoic acid) is one of the natural compounds of CX (Li et al., 2012), and it is a component of CXCTS, Ren-Shen (RS; *Radix Ginseng*; *Panax ginseng C. A. Mey.*) (Chung et al., 2016), LHT, Hou-Po (HP; *Cortex Magnoliae Officinalis*; *Magnolia officinalis Rehder and E. H. Wilson*) (Shim et al., 2015), JWPWS, Bai-Shao (BS; *Radix Paeoniae Alba*; *Paeonia lactiflora Pall.*) (Liu et al., 2015), HQT, Zhi-Zi (ZZ; Fructus Gardeniae; Gardenia jasminoides J. Ellis) (Uddin et al., 2014), and DGLHuiW. Gallic acid also suppresses inflammatory and osteoclast activities (Karatas and Gevrek, 2020). Interestingly, herbal extracts of HP suppresses osteoclastogenesis and bone resorption (Shim et al., 2015). Apigenin (also known as 5,7,4′-Trihydroxyflavone) is one of the natural compounds of Bo-He (BH; *Herba Menthae Haplocalycis*; *Mentha arvensis L.*) (Xu et al., 2017), and it is a component of CXCTS, Chen-Pi (CP; *Pericarpium Citri Reticulatae*; *Citrus reticulata Blanco*) (Brito et al., 2014), JWPWS, HQin (Wang et al., 2018c), HQT, and DGLHuiW. It also inhibits osteoclastogenesis and prevents bone loss (Goto et al., 2015). Caffeic acid is one of the natural compounds of CX (Li et al., 2012), and it is a component of CXCTS, JWPWS, RS (Becker et al., 2015), LHT, DG (Li et al., 2009), DGLHuiW, and GC (Conidi et al., 2019). Caffeic acid shows suppresses bone destruction (Tang et al., 2006). Genistein is one of the natural compounds of ZZ (Wang et al., 2018a), and it is a component of DGLHuiW; it has anti-inflammatory and anti-osteoclastic properties (Lee et al., 2014; Bhattarai et al., 2017). Glycyrrhizic acid is one of the natural compounds of GC (Hennell et al., 2008), and it is a component of CXCTS, LHT, JWPWS, HQT; it suppresses osteoclast differentiation (Yin et al., 2019). Quercetin is one of the natural compounds of DG (Yue et al., 2017), and it is a component of DGLHuiW and GC (Jia et al., 1990). Quercetin induces the apoptosis of osteoclasts, inhibits bone loss, and attenuates cell signaling of tumor necrosis factor receptor family (Pang et al., 2006; Tsuji et al., 2009; Guo et al., 2012).

In this study, the limitations were the lacks of laboratory tests, education, occupation, and lifestyle in the database. However, we found that CHM may reduce risk of overall mortality in patients with osteoporosis or fractures, and may be useful for future investigations in randomized controlled trials (RCT) and functional studies in bone protection. Large-scale RCTs for these CHMs in HIV-infected patients should be performed to determine their relative effectiveness and safety, and to evaluate their potential interactions during regular treatments in these patients.

HIV-infected patients with osteoporosis or fractures who used CHMs as adjunctive therapy had a better survival rate. Based on association rules mining and network analysis, CXCTS, GC, LHT, HQT, JWPWS, and DGLHuiW are potential CHMs for these patients. Further investigations may be undertaken to validate the safety and efficacy of CHMs among these patients. An investigation into the mechanism of actions of the potential compounds of CHMs are required.

## Data Availability

The raw data supporting the conclusion of this article will be made available by the authors, without undue reservation.
