# Supplementary material for: Chinese Herbal Medicine Usage Reduces Overall Mortality in HIV-Infected Patients With Osteoporosis or Fractures
Source: Front Pharmacol. 2021 Apr 16;12:593434. doi: 10.3389/fphar.2021.593434 (PMC8085888; doi:10.3389/fphar.2021.593434)
Supplement: Supplementary file 1 [file datasheet1.docx]

**Supplementary Information**

Supplementary Text

Figures S1-S7

Table S1-S5

**Supplementary Text**

**Figure S1.** **LC-MS/MS analysis of active component standards and the extracts of** **Chuan-Xiong-Cha-Tiao-San (CXCTS)**. (A) Extracted ion chromatogram (EIC) of standards of z-ligustilide and levistolide A. (B) Base peak chromatogram (BPC) and EIC of the extracts of CXCTS.

**Figure S2.** **LC-MS/MS analysis of active component standards and the extracts of** **Liu-He-Tang (LHT)**. (A) Extracted ion chromatogram (EIC) of standards of atractylenolide I. (B) Base peak chromatogram (BPC) and EIC of the extracts of LHT.

**Figure S3. LC-MS/MS analysis of active component standards and the extracts of** **Jia-Wei-Ping-Wei-San (JWPWS)**. (A) Extracted ion chromatogram (EIC) of standards of caffeic acid and hesperidin. (B) Base peak chromatogram (BPC) and EIC of the extracts of JWPWS.

**Figure S4.** **LC-MS/MS analysis of active component standards and the extracts of** **Huang-Qin-Tang (HQT)**. (A) Extracted ion chromatogram (EIC) of standards of baicalein and wogonin. (B) Base peak chromatogram (BPC) and EIC of the extracts of HQT.

**Figure S5. LC-MS/MS analysis of active component standards and the extracts of** **Dang-Gui-Long-Hui-Wan (DGLHuiW)**. (A) Extracted ion chromatogram (EIC) of standards of geniposide and linoleic acid. (B) Base peak chromatogram (BPC) and EIC of the extracts of DGLHuiW.

**Figure S6.** **LC-MS/MS analysis of active component standards and the extracts of** **Gan-Cao (GC) (*Radix Glycyrrhizae Preparata*; *Glycyrrhiza uralensis Fisch.*)**. (A) Extracted ion chromatogram (EIC) of standards of ononin and glycyrrhizic acid. (B) Base peak chromatogram (BPC) and EIC of the extracts of GC.

**Figure S7.** **Network analysis for CHM prescription pattern in HIV-infected patients with osteoporosis or fractures (with dosage).** The lines connecting CHMs represent the support value: thicker lines represent higher support values (Support (X) (%)). The line color between CHMs shows the lift value: darker lines represent stronger connections with higher lift values. The red circle represents herbal formulas, while the green circle represents single herbs. The size of the circle for each CHM shows its dosage: larger circles indicate higher dosage (Table 3). Support (X) (%) = Frequency of prescriptions of X and Y products / total prescriptions x 100%. Lift = Confidence (X →Y) (%) / P (Y) (%). Confidence (X →Y) (%) = Frequency of prescriptions of X and Y products / Frequency of prescriptions of X product x 100%. P (Y) (%) = Frequency of prescriptions of Y product / total prescriptions x 100%. Abbreviations: CHM, Chinese herbal medicine; CXCTS, Chuan-Xiong-Cha-Tiao-San; GC: Gan-Cao; LHT: Liu-He-Tang; HQT: Huang-Qin-Tang; JWPWS: Jia-Wei-Ping-Wei-San; DGLHuiW, Dang-Gui-Long-Hui-Wan.

**Table S1.** Composition of the most commonly used herbal formulas and single herbs for HIV infected patients with osteoporosis or with fractures in Taiwan.

**Table S2.** Demographic characteristics (time span and income) of HIV-infected patients with osteoporosis or fractures according to Chinese herbal medicine usage in Taiwan.

**Table S3.** Distribution of the cumulative days of CHM treatment during the study period among HIV-infected patients with osteoporosis or fractures.

**Table S4.** Numbers and parameters for counting the cumulative incidence of overall mortality at figure 3 for non-CHM users.

**Table S5.** Numbers and parameters for counting the cumulative incidence of overall mortality at figure 3 for CHM users.

**Figure. S1 A**

**Standard reference compounds for Chuan-Xiong-Cha-Tiao-San (CXCTS)**

**
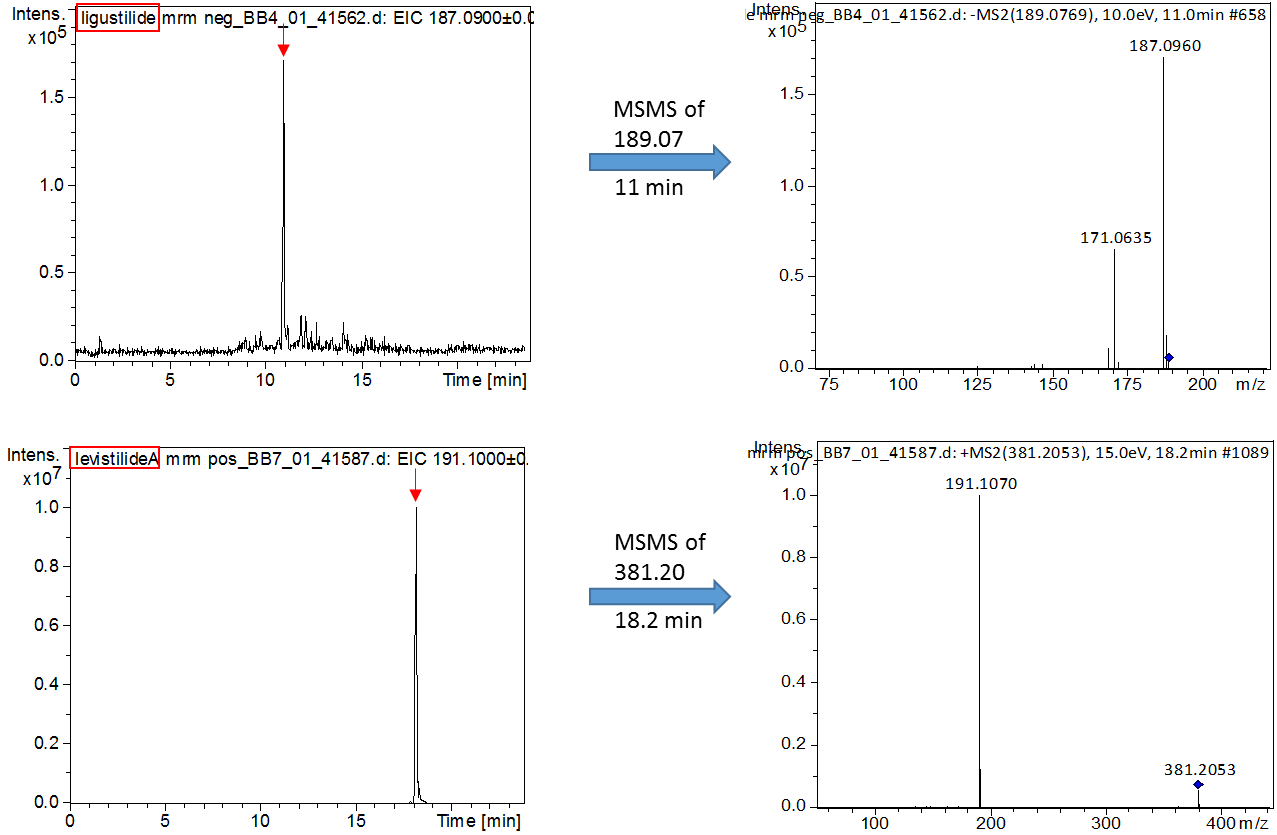
**

**Figure. S1 B**

**Chuan-Xiong-Cha-Tiao-San (CXCTS) extract**

**
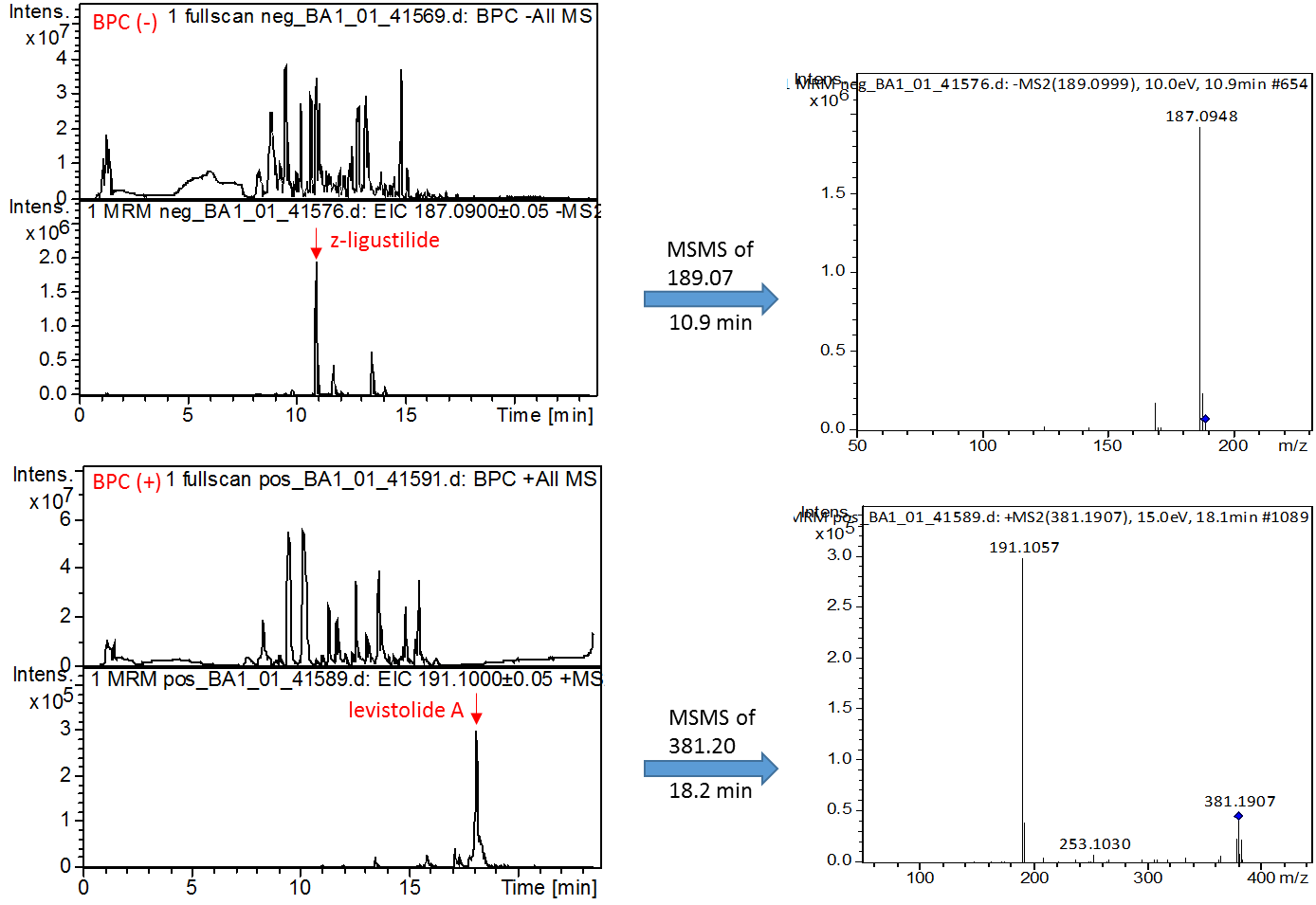
**

**Figure. S2 A**

**Standard reference compounds for Liu-He-Tang (LHT)**

**
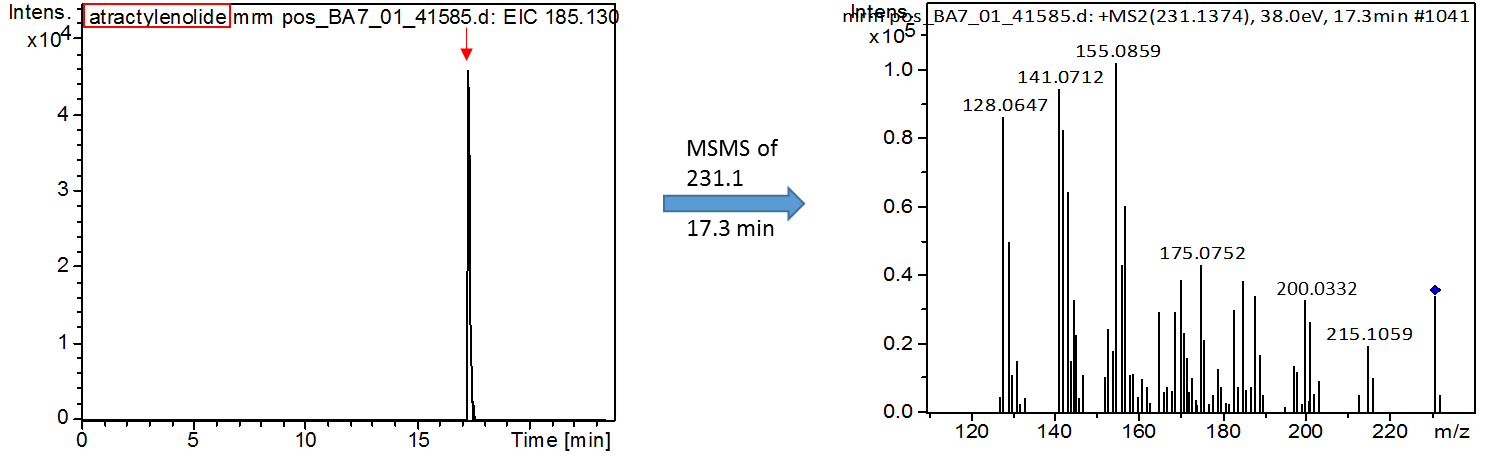
**

**Figure. S2 B**

**
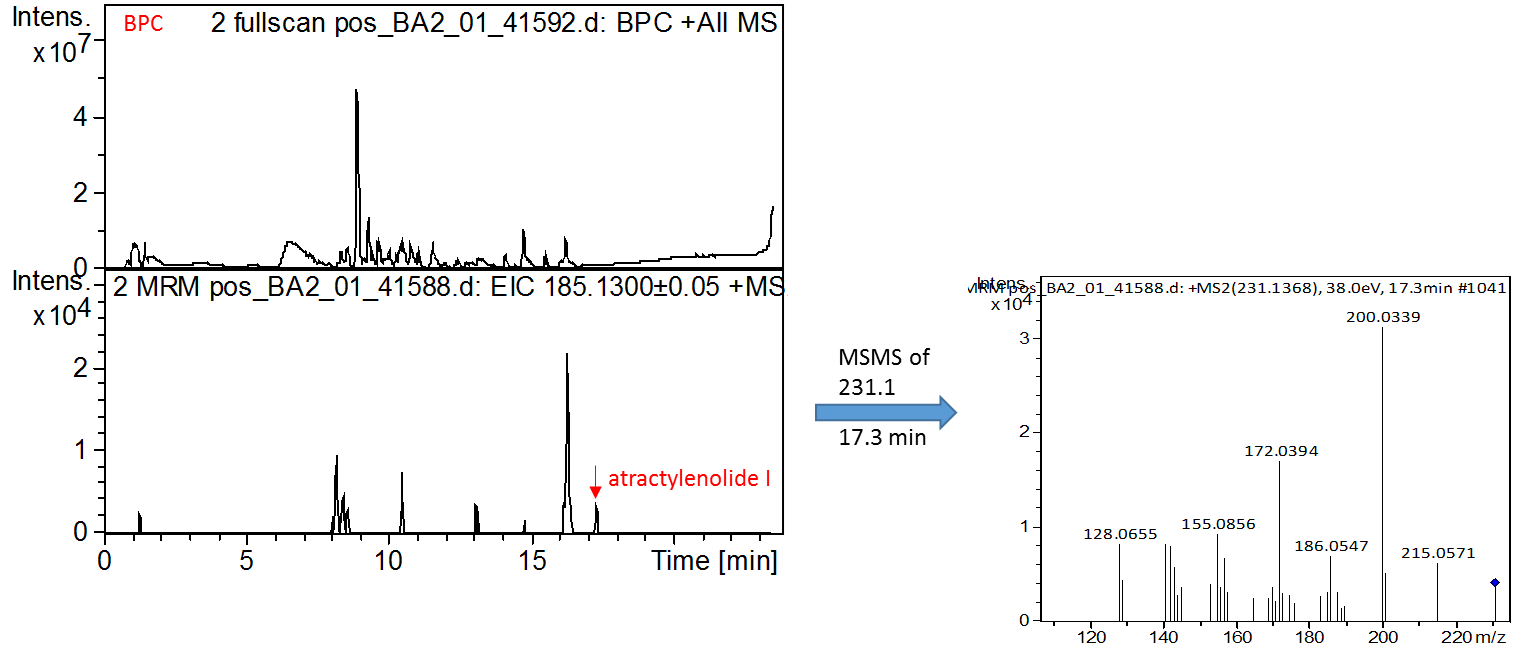
Liu-He-Tang (LHT) extract**

**Figure. S3 A**

**Standard reference compounds for Jia-Wei-Ping-Wei-San (JWPWS)**

**
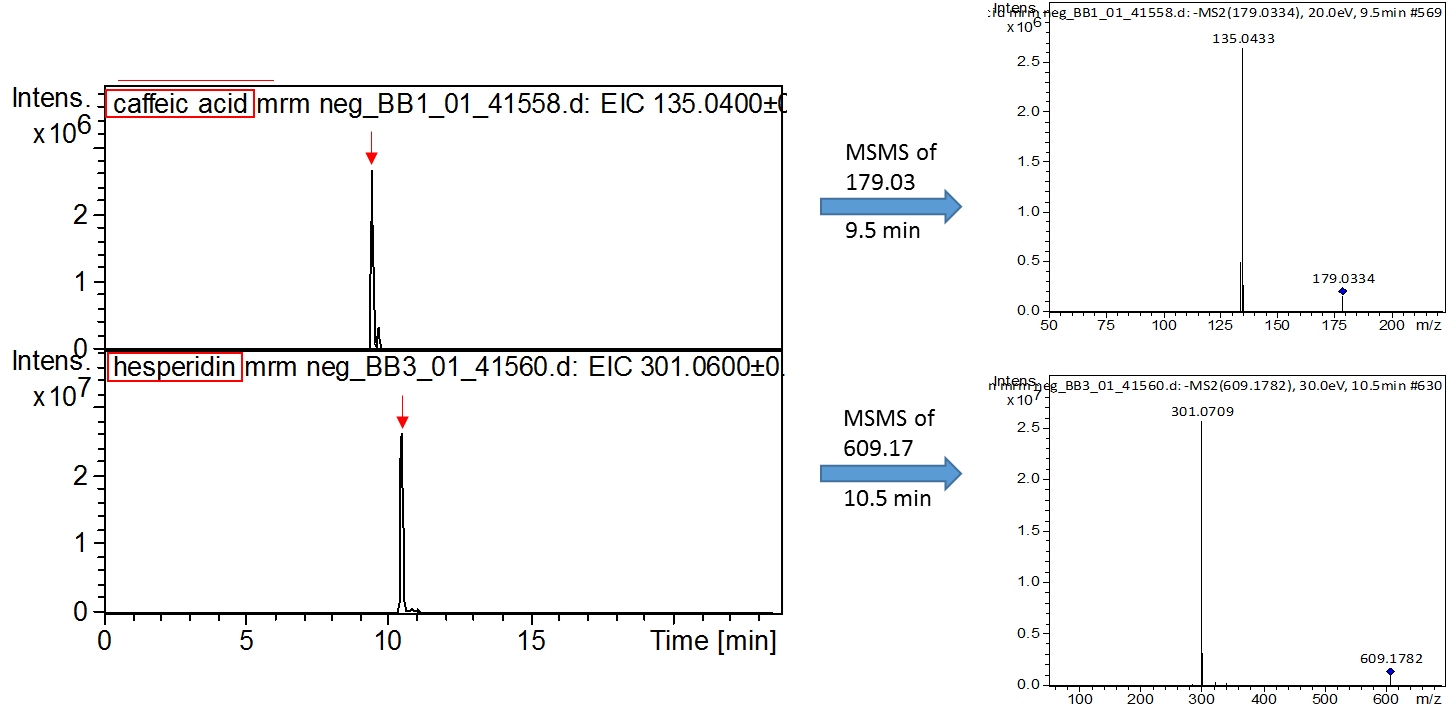
**

**Figure. S3 B**

**Jia-Wei-Ping-Wei-San (JWPWS) extract**


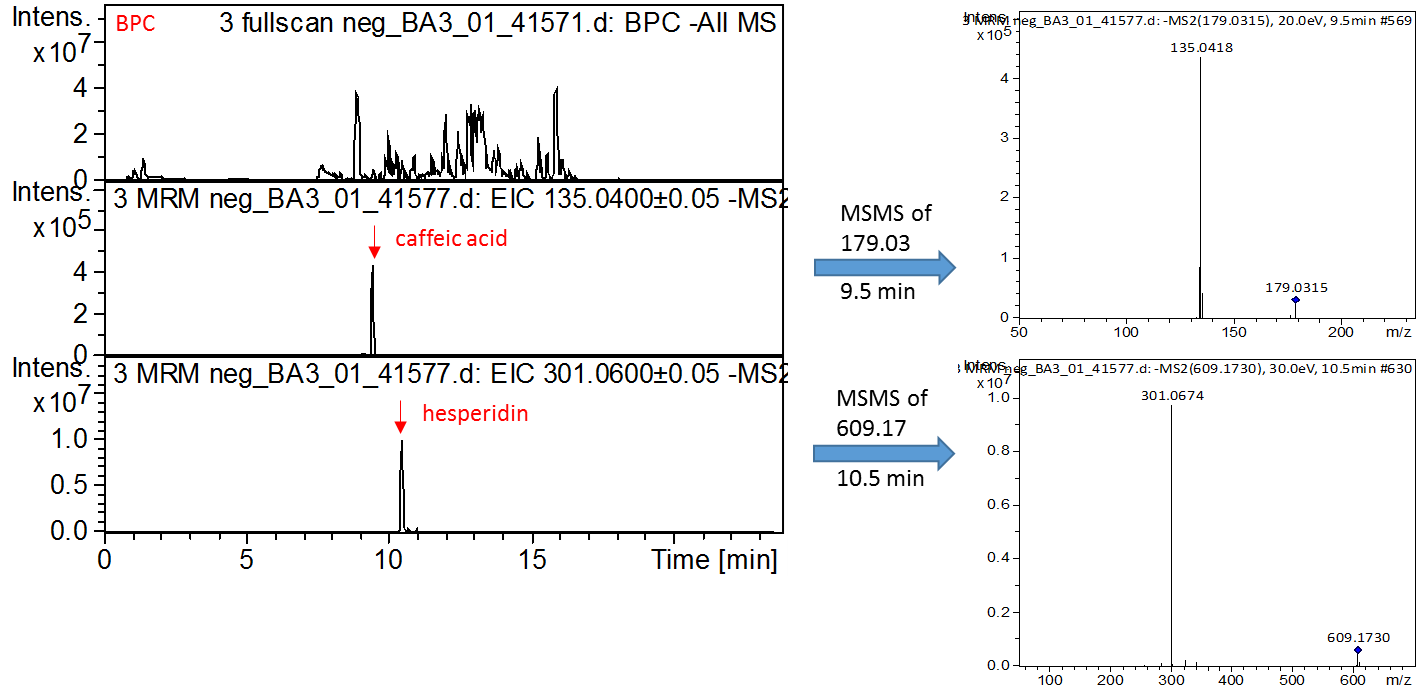


**Figure. S4 A**

**Standard reference compounds for Huang-Qin-Tang (HQT)**


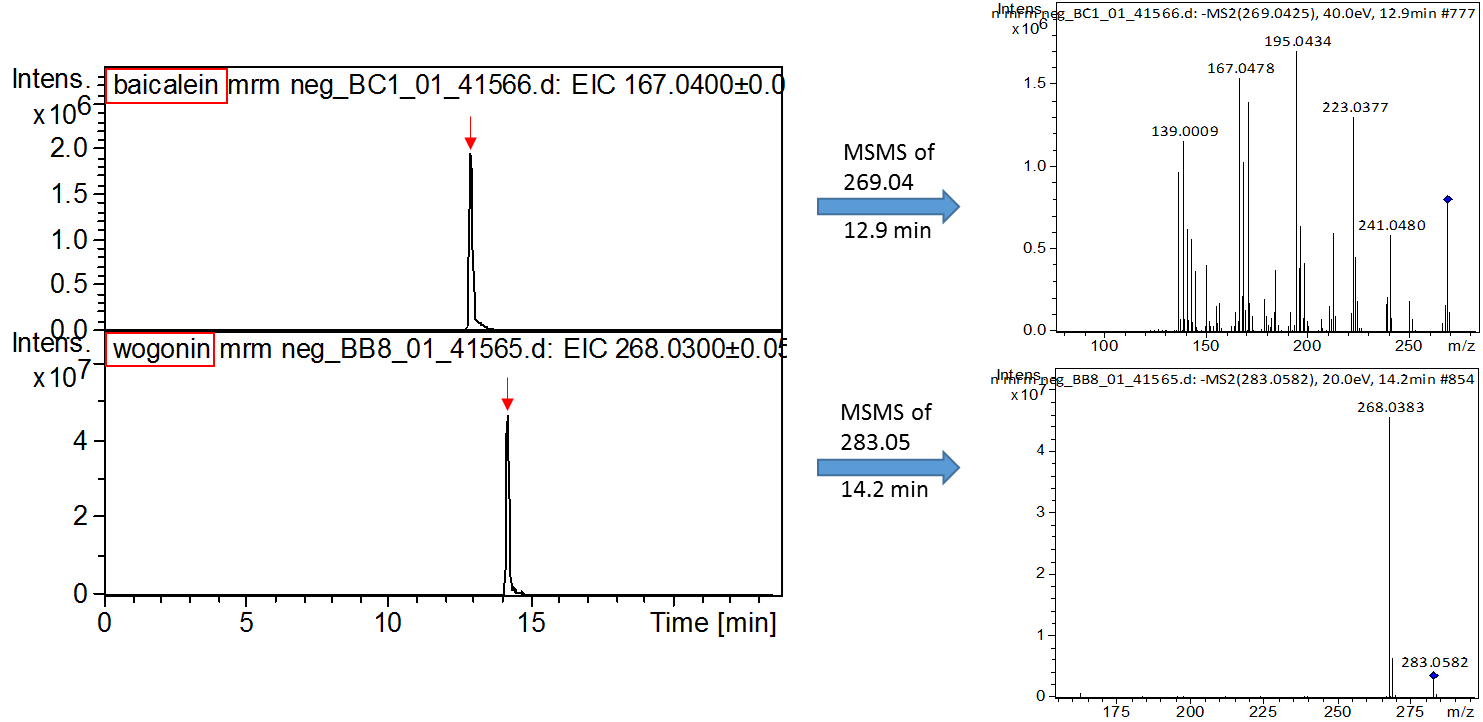


**Figure. S4 B**

**Huang-Qin-Tang (HQT) extract**


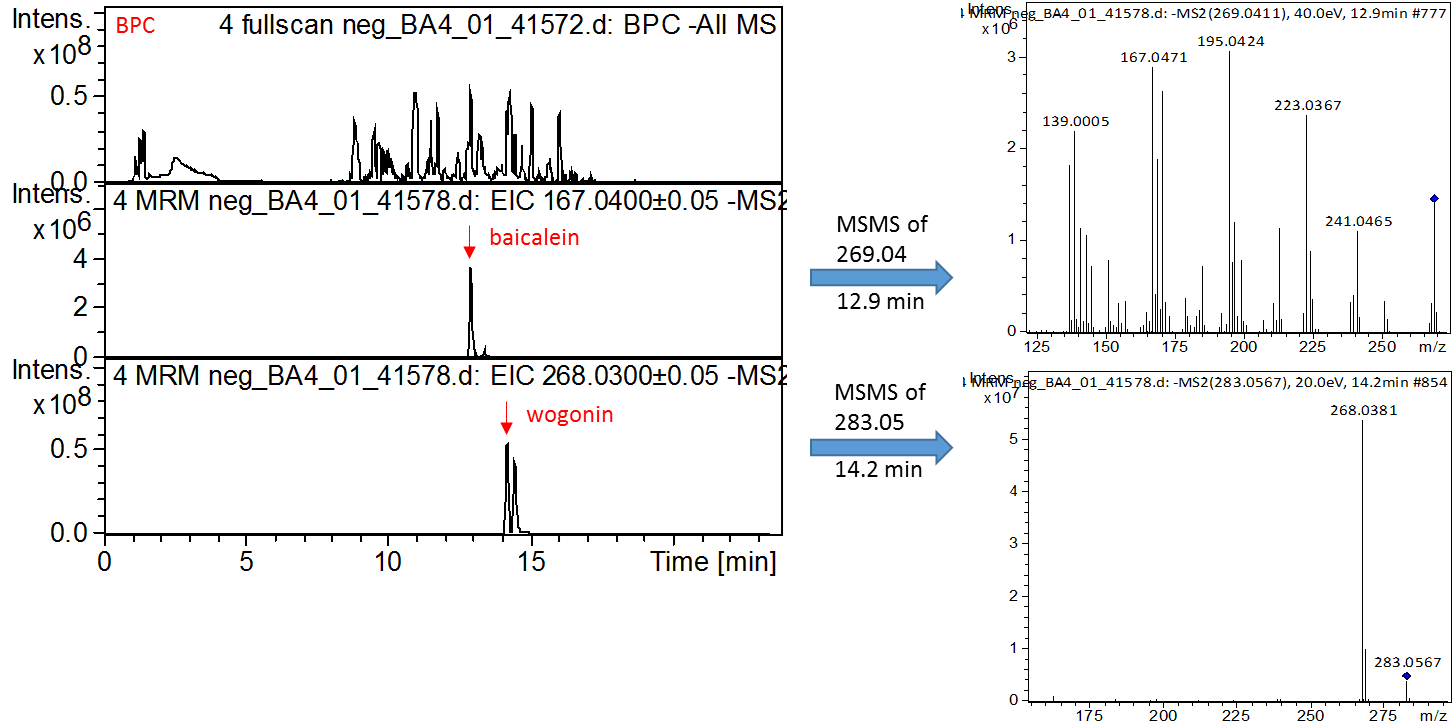


**Figure. S5 A**

**Standard reference compounds for Dang-Gui-Long-Hui-Wan (DGLHuiW)**

**
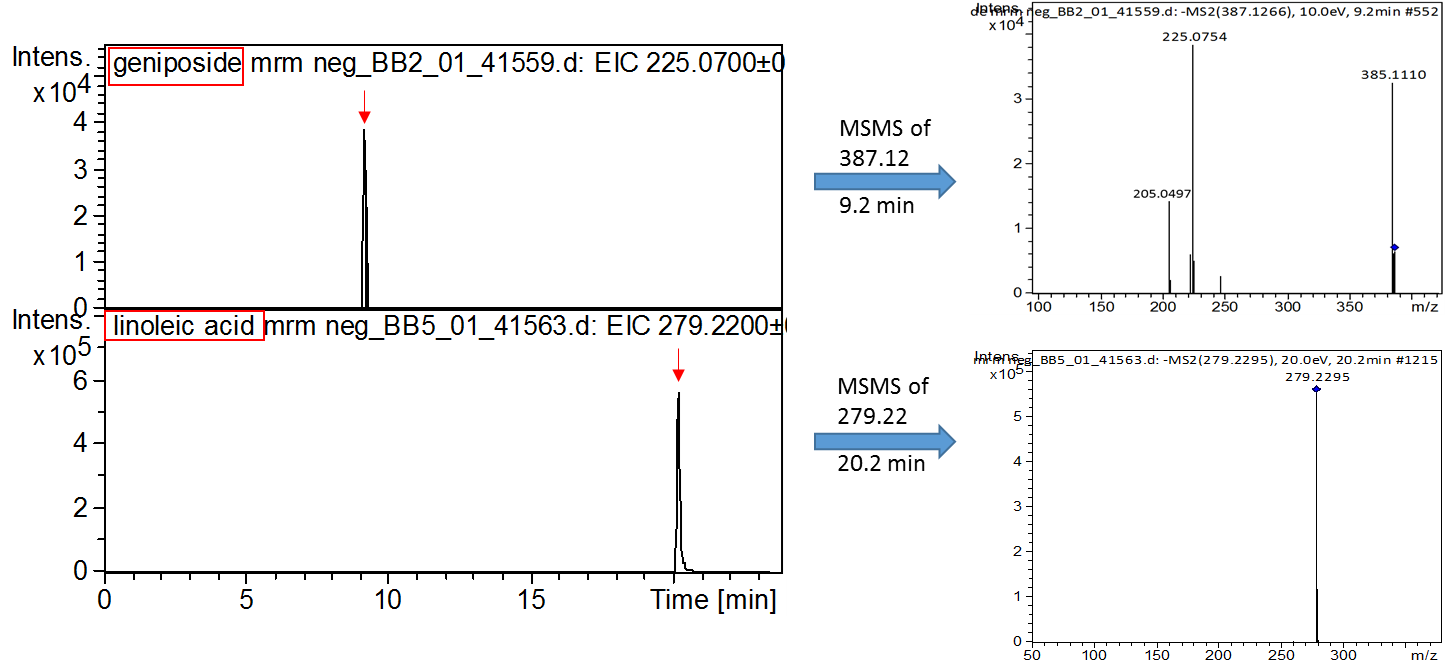
**

**Figure. S5 B**

**Dang-Gui-Long-Hui-Wan (DGLHuiW) extract**


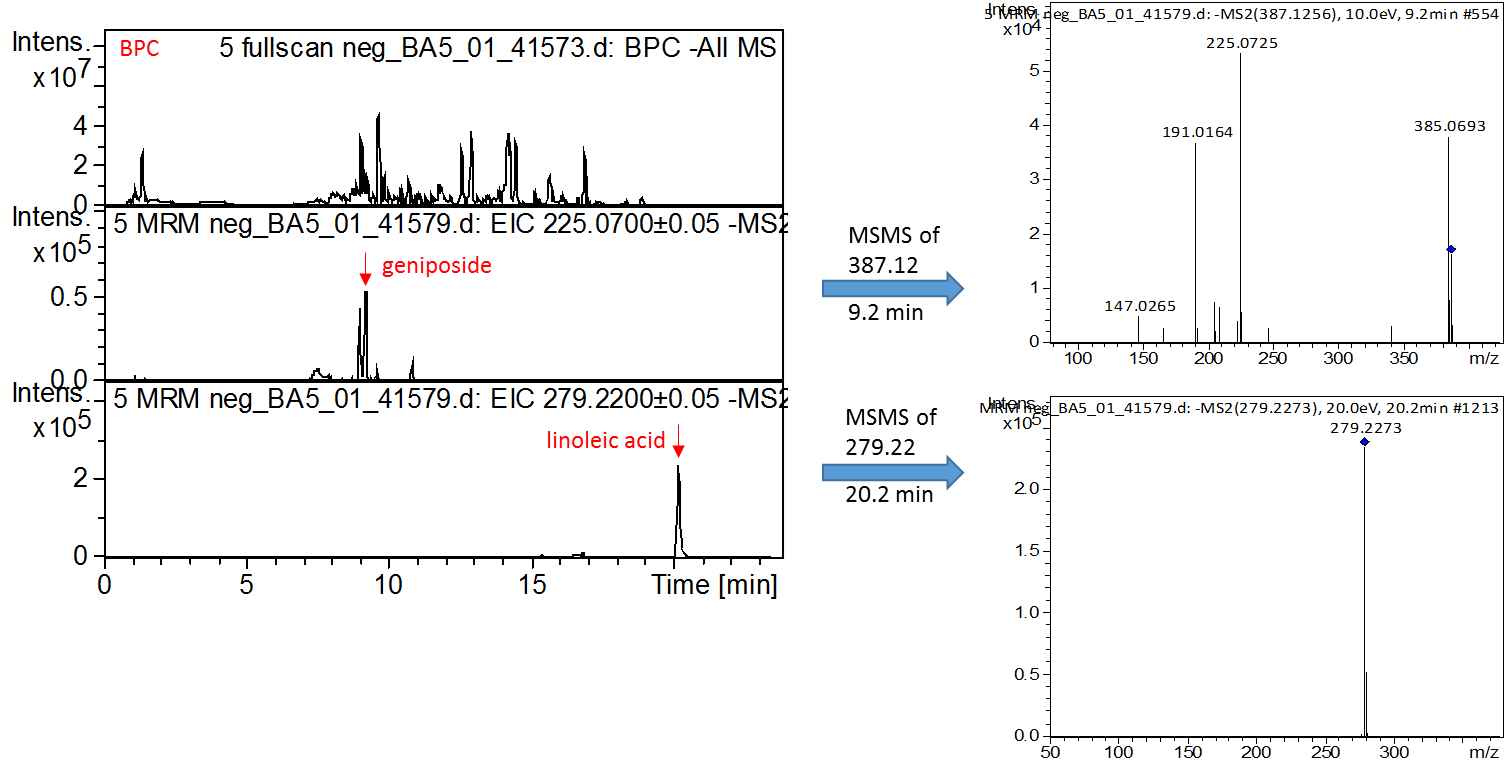


**Figure. S6 A**

**Standard reference compounds for Gan-Cao (GC) (*Radix Glycyrrhizae Preparata*; *Glycyrrhiza uralensis Fisch.*)**


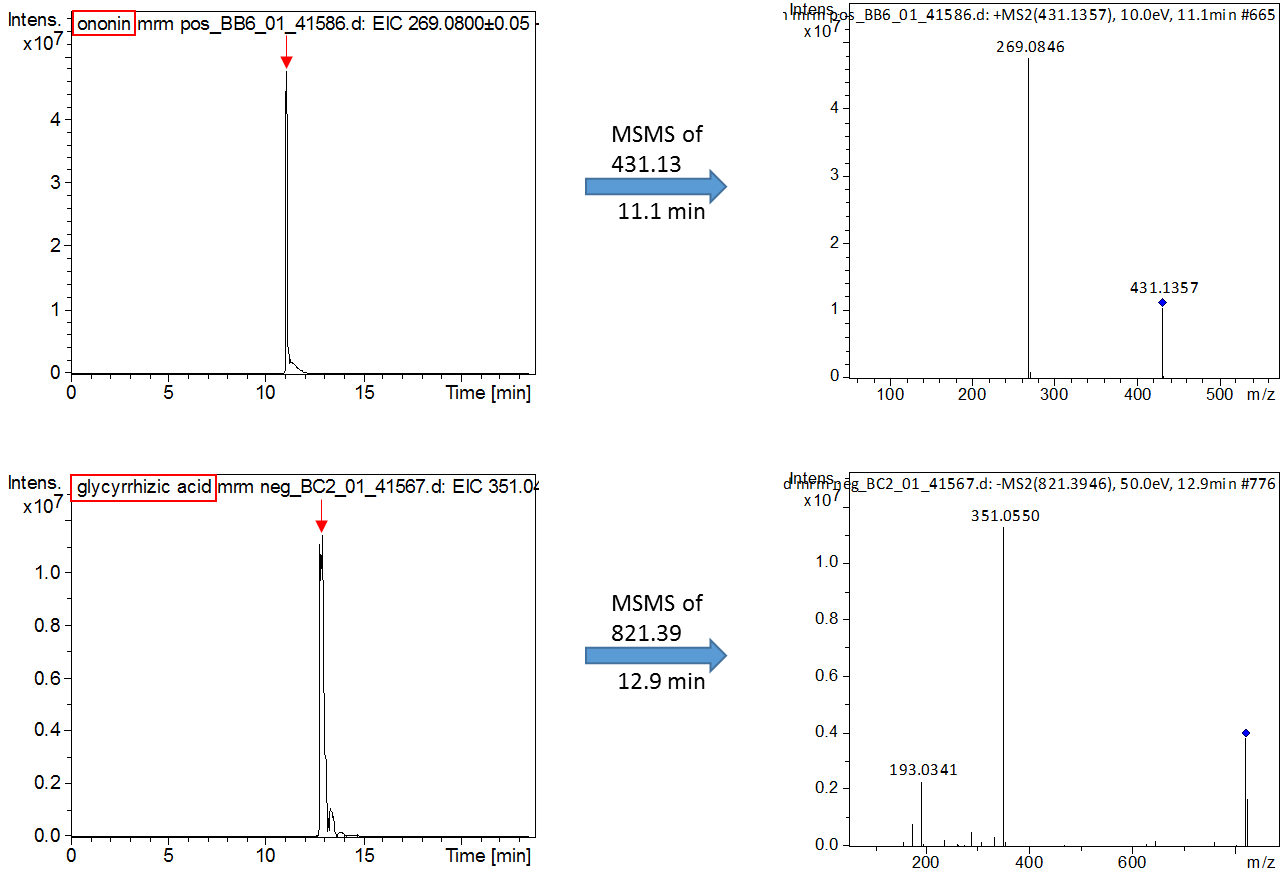


**Figure. S6 B**

**Gan-Cao (GC) (*Radix Glycyrrhizae Preparata*; *Glycyrrhiza uralensis Fisch.*) extract**


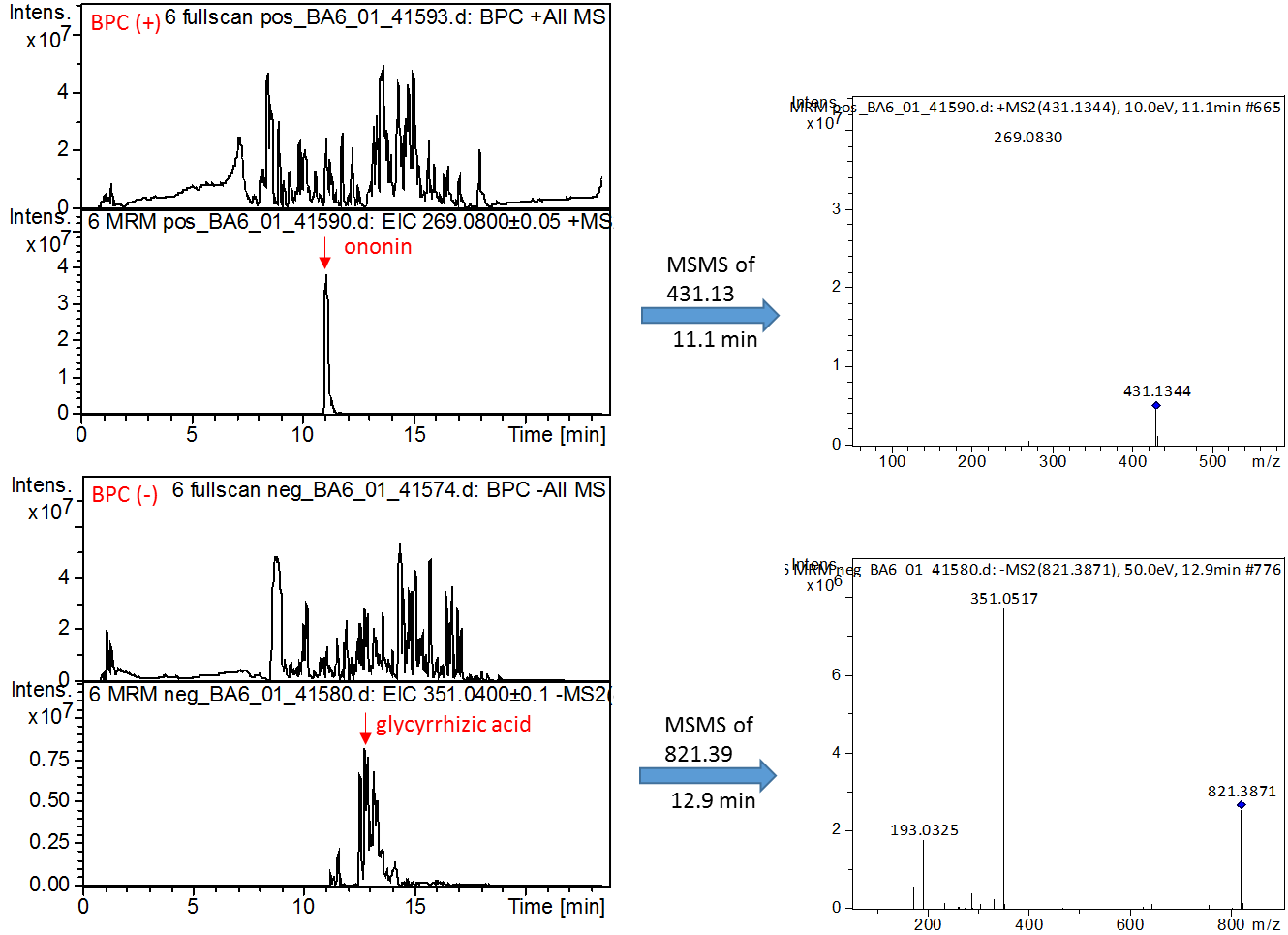


**Figure. S7**


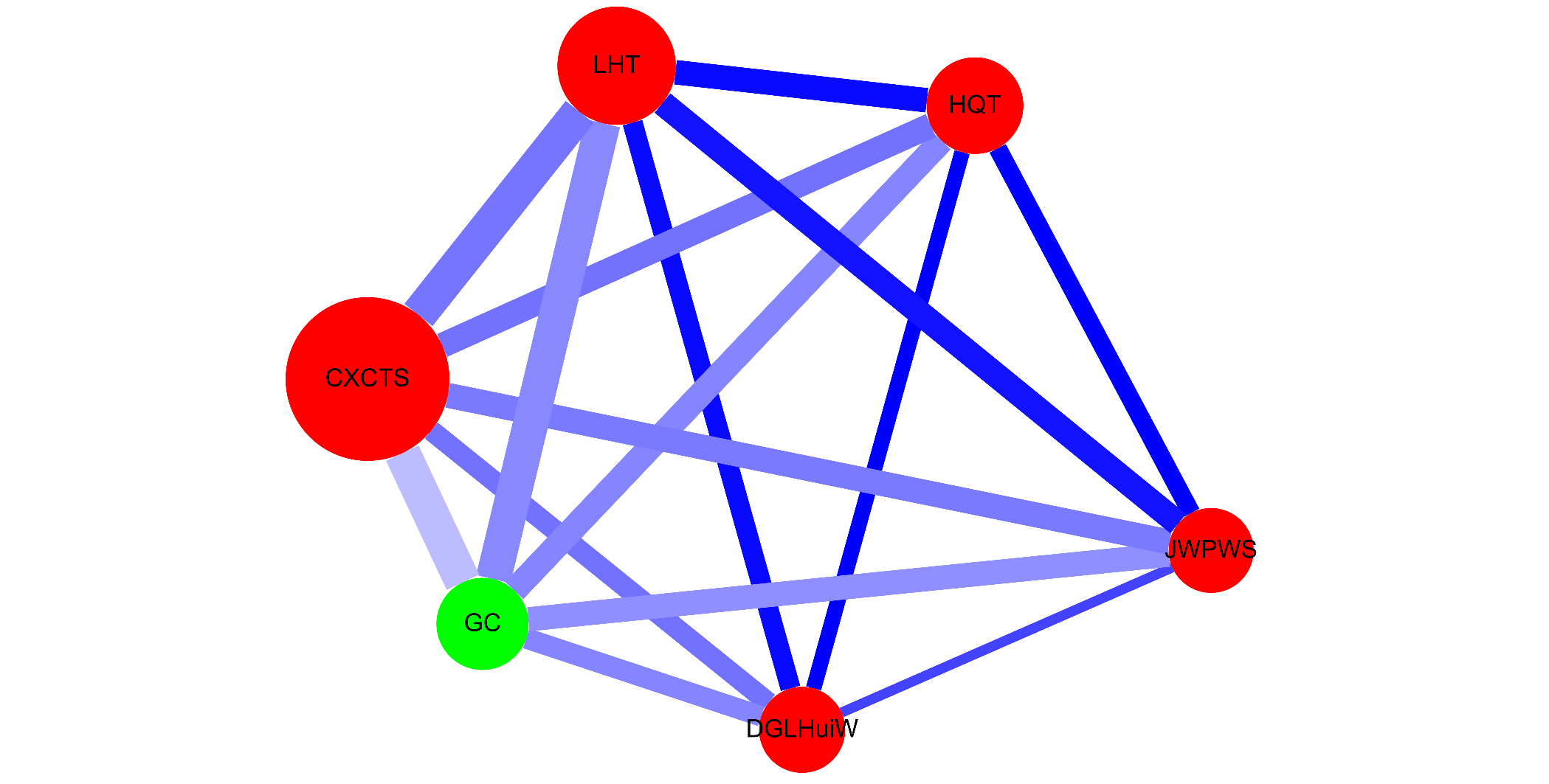


| **TABLE S1 \|** Composition of the most commonly used herbal formulas and single herbs for HIV infected patients with osteoporosis or with fractures in Taiwan. | | | | | | | | | | |
| --- | --- | --- | --- | --- | --- | --- | --- | --- | --- | --- |
| **Formulas** |  | **Chinese name** | **Total number of herbs** | **Number** | **Composition (Pin-yin name (latin name; botanical plant name))** | **Frequency of prescriptions** | **Person-year** | **Percentage of usage person** | **Average drug dose per day (g)** | **Average duration for prescription (days)** |
| **Total** |  |  |  |  |  | **3745** | **722.9** | **100** | **13.4** | **6.7** |
| **Herbal formula (Pin-yin name)** |  |  |  |  |  | **3638** | **722.9** | **100** | **10.5** | **6.6** |
|  | Chuan-Xiong-Cha-Tiao-San (CXCTS) | 川芎茶調散 | 10 | 1 | Bo-He (*Herba Menthae Haplocalycis*; *Mentha arvensis L.*) | 311 | 183.7 | 26.2 | 5.3 | 7.3 |
|  |  |  |  | 2 | Chuan-Xiong (*Rhizoma Chuanxiong*; *Ligusticum sinense Oliv.*) |  |  |  |  |  |
|  |  |  |  | 3 | Bai-Zhi (*Radix Angelicae Dahuricae*; *Angelica dahurica (Hoffm.) Benth. & Hook.f. ex Franch. & Sav.*) |  |  |  |  |  |
|  |  |  |  | 4 | Qiang-Huo (*Rhizoma seu Radix Notopterygii*; *Notopterygium forbesii var. oviforme (Shan) H.T. Chang*) |  |  |  |  |  |
|  |  |  |  | 5 | Xi-Xin (*Herba cum Radix Asari*; *Asarum sieboldii Miq.*) |  |  |  |  |  |
|  |  |  |  | 6 | Xiang-Fu (*Rhizoma Cyperi*; *Cyperus rotundus L.*) |  |  |  |  |  |
|  |  |  |  | 7 | Jing-Jie (*Herba Schizonepetae*; *Schizonepeta tenuifolia (Benth.) Briq.*) |  |  |  |  |  |
|  |  |  |  | 8 | Fang-Feng (*Radix Saposhnikoviae*; *Saposhnikovia divaricata (Turcz.) Schischk.*) |  |  |  |  |  |
|  |  |  |  | 9 | Gan-Cao (*Radix Glycyrrhizae Preparata*; *Glycyrrhiza uralensis Fisch.*) |  |  |  |  |  |
|  |  |  |  | 10 | Lu-Cha (*Folium Camelliae Sinensis*; *Camellia sinensis (L.) Kuntze*) |  |  |  |  |  |
|  | Liu-He-Tang (LHT) | 六和湯 | 14 | 1 | Ren-Shen (*Radix Ginseng*; *Panax ginseng C.A.Mey.*) | 175 | 18 | 2.7 | 4.3 | 8 |
|  |  |  |  | 2 | Sha-Ren ( *Fructus Amomi*; *Amomum villosum Lour.*) |  |  |  |  |  |
|  |  |  |  | 3 | Zhi-Ban-Xia ( *Rhizoma Pinelliae Preparatum*; *Pinellia ternata (Thunb.) Makino*) |  |  |  |  |  |
|  |  |  |  | 4 | Xing-Ren (*Semen Armeniacae*; *Prunus armeniaca L.*) |  |  |  |  |  |
|  |  |  |  | 5 | Bai-Zhu (*Rhizoma Atractylodis*; *Atractylodes macrocephala Koidz.*) |  |  |  |  |  |
|  |  |  |  | 6 | Huo-Xiang ( *Herba Agastaches*; *Agastache rugosa (Fisch. & C.A.Mey.) Kuntze*) |  |  |  |  |  |
|  |  |  |  | 7 | Bai-Bian-Dou (*Semen Lablab Album*; *Dolichos lablab L*.) |  |  |  |  |  |
|  |  |  |  | 8 | Chi-Fu-Ling (*Poria*; *Wolfiporia extensa (Peck) Ginns*) |  |  |  |  |  |
|  |  |  |  | 9 | Mu-Gua (*Fructus Chaenomelis*; *Chaenomeles speciosa (Sweet) Nakai*) |  |  |  |  |  |
|  |  |  |  | 10 | Hou-Po (*Cortex Magnoliae Officinalis*; *Magnolia officinalis Rehder & E.H.Wilson*) |  |  |  |  |  |
|  |  |  |  | 11 | Xiang-Ru ( *Herba Moslae*; *Elsholtzia splendens Nakai ex F.Maek*.) |  |  |  |  |  |
|  |  |  |  | 12 | Zhi-Gan-Cao (*Radix Glycyrrhizae Preparata*; *Glycyrrhiza uralensis Fisch*.) |  |  |  |  |  |
|  |  |  |  | 13 | Sheng-Jiang (*Rhizoma Zingiberis Recens*; *Zingiber officinale Roscoe*) |  |  |  |  |  |
|  |  |  |  | 14 | Da-Zao (*Fructus Jujube*; *Ziziphus jujuba Mill.*) |  |  |  |  |  |
|  | Jia-Wei-Ping-Wei-San (JWPWS) | 加味平胃散 | 11 | 1 | Cang-Zhu ( *Rhizoma Atractylodis* ; *Atractylodes lancea (Thunb.) DC.*) | 131 | 29.5 | 2.7 | 1.5 | 8.1 |
|  |  |  |  | 2 | Mai-Ya (*Fructus Hordei Germinatus*; *Hordeum vulgare L.*) |  |  |  |  |  |
|  |  |  |  | 3 | Hou-Po (*Cortex Magnoliae Officinalis*; *Magnolia officinalis Rehder & E.H.Wilson*) |  |  |  |  |  |
|  |  |  |  | 4 | Chen-Pi (*Pericarpium Citri Reticulatae*; *Citrus reticulata Blanco*) |  |  |  |  |  |
|  |  |  |  | 5 | Qing-Pi ( *Pericarpium Citri Reticulate Viride*; *Citrus reticulata Blanco*) |  |  |  |  |  |
|  |  |  |  | 6 | Chuan-Xiong (*Rhizoma Chuanxiong*; *Ligusticum sinense Oliv.*) |  |  |  |  |  |
|  |  |  |  | 7 | Sha-Ren (*Fructus Amomi*; *Amomum villosum Lour.*) |  |  |  |  |  |
|  |  |  |  | 8 | Xiang-Fu (*Rhizoma Cyperi*; *Cyperus rotundus L.*) |  |  |  |  |  |
|  |  |  |  | 9 | Gan-Cao (*Radix Glycyrrhizae Preparata*; *Glycyrrhiza uralensis Fisch.*) |  |  |  |  |  |
|  |  |  |  | 10 | Da-Zao (*Fructus Jujube*; *Ziziphus jujuba Mill*.) |  |  |  |  |  |
|  |  |  |  | 11 | Sheng-Jiang (*Rhizoma Zingiberis Recens*; *Zingiber officinale Roscoe*) |  |  |  |  |  |
|  | Huang-Qin-Tang (HQT) | 黃芩湯 | 4 | 1 | Huang-Qin (*Radix Scutellariae*; *Scutellaria baicalensis Georgi*) | 124 | 6.8 | 0.7 | 3 | 8.5 |
|  |  |  |  | 2 | Bai-Shao (*Radix Paeoniae Alba*; *Paeonia lactiflora Pall.*) |  |  |  |  |  |
|  |  |  |  | 3 | Zhi-Gan-Cao (*Radix Glycyrrhizae Preparata*; *Glycyrrhiza uralensis Fisch.*) |  |  |  |  |  |
|  |  |  |  | 4 | Da-Zao (*Fructus Jujube*; *Ziziphus jujuba Mill*.) |  |  |  |  |  |
|  | Dang-Gui-Long-Hui-Wan (DGLHuiW) | 當歸龍薈丸 | 12 | 1 | Dang-Gui (*Radix Angelicae Sinensi*; *Angelica sinensis (Oliv.) Diels*) | 105 | 6.8 | 0.7 | 1.9 | 8.8 |
|  |  |  |  | 2 | Long-Dan-Cao (*Radix Gentianae*; *Gentiana scabra Bunge*) |  |  |  |  |  |
|  |  |  |  | 3 | Zhi-Zi (*Fructus Gardeniae*; *Gardenia jasminoides J.Ellis*) |  |  |  |  |  |
|  |  |  |  | 4 | Huang-Lian (*Rhizoma Coptidis*; *Coptis chinensis Franch.*) |  |  |  |  |  |
|  |  |  |  | 5 | Huang-Bai (*Cortex Phellodendri*; *Phellodendron amurense Rupr.*) |  |  |  |  |  |
|  |  |  |  | 6 | Huang-Qin (*Radix Scutellariae*; *Scutellaria baicalensis Georgi*) |  |  |  |  |  |
|  |  |  |  | 7 | Lu-Hui (*Aloe*; *Aloe barbadensis Mill.*) |  |  |  |  |  |
|  |  |  |  | 8 | Da-Huang (*Radix et Rhizoma Rhei*; *Rheum palmatum L*.) |  |  |  |  |  |
|  |  |  |  | 9 | Qing-Dai (*Indigo Naturalis*; *Baphicacanthus cusia (Nees) Bremek.*) |  |  |  |  |  |
|  |  |  |  | 10 | Mu-Xiang (*Radix Aucklandiae*; *Vladimiria souliei (Franch.) Ling*) |  |  |  |  |  |
|  |  |  |  | 11 | Sheng-Jiang (*Rhizoma Zingiberis Recens*; *Zingiber officinale Roscoe*) |  |  |  |  |  |
|  |  |  |  | 12 | She-Xiang (*Moschus*) |  |  |  |  |  |
| **Single herbs (Pin-yin name)** |  |  |  |  |  | **3035** | **709** | **97.3** | **3.9** | **6.7** |
|  | Gan-Cao (GC) | 甘草 | 1 | 1 | **Gan-Cao** (*Radix Glycyrrhizae Preparata; Glycyrrhiza uralensis Fisch.*) | 360 | 198.4 | 26.2 | 1 | 7.1 |
| *Sorted by frequency of prescriptions. | | | | | | | | | | |
| Information are obtained from the websites (http://www.americandragon.com/index.htm; http://old.tcmwiki.com/; http://www.shen-nong.com/eng/front/index.html; http://www.ipni.org/; http://www.theplantlist.org/). | | | | | | | | | | |

| **TABLE S2 \|** Demographic characteristics (time span and income) of HIV-infected patients with osteoporosis or fractures according to Chinese herbal medicine usage in Taiwan. | | | | | | |
| --- | --- | --- | --- | --- | --- | --- |
| **Characteristics** | **Total subjects** | | ***p*-value** | **Matched subjects** | | ***p*-value** |
|  | **CHM users (N=160)** | **Non-CHM users (N=338)** |  | **CHM users (N=149)** | **Non-CHM users (N=149)** |  |
|  | **N (%)** | **N (%)** |  | **N (%)** | **N (%)** |  |
| **Time span between the fractures and the start of the CHM treatment (day; Mean±SD)** | 77.18± 83.66 | NA | NA | 78.55± 84.49 | NA | NA |
|  |  |  |  |  |  |  |
| **Income (new Taiwan dollars; Mean±SD)** | NT$13,910± 18,151 | NT$14586± 20282 | 0.720 | NT$16059± 20704 | NT$15191± 19264 | 0.708 |
| N, number; CHM, Chinese herbal medicine; SD, standard deviation; NA, not applicable. | | | | | | |
| *p*-values were obtained by the un-paired Student t test. | | | | | | |
| Time span between the fractures and the start of the CHM treatment was from the diagnosed date of osteoporosis or fractures to the start of the CHM treatment (day; Mean±SD). | | | | | | |

| **TABLE S3 \|** Distribution of the cumulative days of CHM treatment during the study period among HIV-infected patients with osteoporosis or fractures. | | | |
| --- | --- | --- | --- |
| **Cumulative CHM treatment days within the first year after osteoporosis or fractures** | **Cumulative CHM treatment days during the study period (started after the index date)** | **CHM users** | |
|  |  | **N** | **%** |
| **≥14 days (N = 149)** |  |  | |
|  | **day < 180** | 105 | 70.5 |
|  | **180 ≤ day< 360** | 28 | 18.8 |
|  | **day ≥ 360** | 16 | 10.7 |
| **≥28 days (N =89)** |  |  |  |
|  | **day < 180** | 54 | 60.7 |
|  | **180 ≤ day< 360** | 21 | 23.6 |
|  | **day ≥ 360** | 14 | 15.7 |
| **≥56 days (N = 48)** |  |  |  |
|  | **day < 180** | 21 | 43.8 |
|  | **180 ≤ day< 360** | 14 | 29.2 |
|  | **day ≥ 360** | 13 | 27.1 |
| **≥84 days (N = 27)** |  |  |  |
|  | **day < 180** | 8 | 29.6 |
|  | **180 ≤ day< 360** | 8 | 29.6 |
|  | **day ≥ 360** | 11 | 40.7 |
| N, number; CHM, Chinese herbal medicine. | | | |
| The index date of this study was from the day on which the 14, 28, 56, or 84 cumulative days of CHM treatment with the first year were completed. | | | |
| Cumulative CHM treatment days during the study period was started after the index date. | | | |

| **TABLE S4 \|** Numbers and parameters for counting the cumulative incidence of overall mortality at figure 3 for non-CHM users | | | | | | | | |
| --- | --- | --- | --- | --- | --- | --- | --- | --- |
| **Interval** | | **Effective Sample Size (n)** | **Number of failed (NF)** | **Number of censored (NC)** | **Conditional probability of failure (q)** | **1-conditional probability of failure (1-q)** | **Survival (p)** | **Overall mortality (1-Survival)** |
| **Lower** | **Upper** |  |  |  |  |  |  |  |
| 0 | 1 | 146 | 9 | 6 | 0.0616 | 0.9384 | 1 | 0 |
| 1 | 2 | 122.5 | 4 | 23 | 0.0327 | 0.9673 | 0.9384 | 0.0616 |
| 2 | 3 | 99.5 | 2 | 15 | 0.0201 | 0.9799 | 0.9077 | 0.0923 |
| 3 | 4 | 85 | 4 | 10 | 0.0471 | 0.9529 | 0.8895 | 0.1105 |
| 4 | 5 | 69.5 | 7 | 13 | 0.1007 | 0.8993 | 0.8476 | 0.1524 |
| 5 | 6 | 48 | 4 | 16 | 0.0833 | 0.9167 | 0.7622 | 0.2378 |
| 6 | 7 | 30.5 | 2 | 11 | 0.0656 | 0.9344 | 0.6987 | 0.3013 |
| 7 | 8 | 19.5 | 2 | 7 | 0.1026 | 0.8974 | 0.6529 | 0.3471 |
| 8 | 9 | 11 | 0 | 6 | 0 | 1 | 0.5859 | 0.4141 |
| 9 | 10 | 7 | 0 | 2 | 0 | 1 | 0.5859 | 0.4141 |
| 10 | 11 | 5.5 | 0 | 1 | 0 | 1 | 0.5859 | 0.4141 |
| 11 | 12 | 4 | 0 | 2 | 0 | 1 | 0.5859 | 0.4141 |
| 12 | 13 | 2 | 1 | 2 | 0.5 | 0.5 | 0.5859 | 0.4141 |
| Interval (Lower; Upper) means the follow-up time; for example: lower=0; upper=1 means the interval between 0-1 year. | | | | | | | | |
| Effective sample size (n) means the total sample number in the non-CHM users. n = N-1/2 (NC). | | | | | | | | |
| Number of censored (NC) means the number of withdrawal or loss during the interval in the non-CHM users in this study. | | | | | | | | |
| Number of failed (NF) means the number of death during the interval in the non-CHM users in this study. | | | | | | | | |
| Conditional probability of failure (q) = NF/n. | | | | | | | | |
| Survival (p) = ㄇp = ㄇ(1-q). | | | | | | | | |
| Overall mortality = 1-Survival. | | | | | | | | |

| **TABLE S5 \|** Numbers and parameters for counting the cumulative incidence of overall mortality at figure 3 for CHM users | | | | | | | | |
| --- | --- | --- | --- | --- | --- | --- | --- | --- |
| **Interval** | | **Effective Sample Size (n)** | **Number of failed (NF)** | **Number of censored (NC)** | **Conditional probability of failure (q)** | **1-conditional probability of failure (1-q)** | **Survival (p)** | **Overall mortality (1-Survival)** |
| **Lower** | **Upper** |  |  |  |  |  |  |  |
| 0 | 1 | 145.5 | 3 | 7 | 0.0206 | 0.9794 | 1 | 0 |
| 1 | 2 | 129.5 | 4 | 19 | 0.0309 | 0.9691 | 0.9794 | 0.0206 |
| 2 | 3 | 107.5 | 0 | 17 | 0 | 1 | 0.9491 | 0.0509 |
| 3 | 4 | 91 | 5 | 16 | 0.0549 | 0.9451 | 0.9491 | 0.0509 |
| 4 | 5 | 71.5 | 3 | 13 | 0.042 | 0.958 | 0.897 | 0.103 |
| 5 | 6 | 55 | 0 | 14 | 0 | 1 | 0.8593 | 0.1407 |
| 6 | 7 | 42.5 | 2 | 11 | 0.0471 | 0.9529 | 0.8593 | 0.1407 |
| 7 | 8 | 32 | 0 | 6 | 0 | 1 | 0.8189 | 0.1811 |
| 8 | 9 | 24 | 0 | 10 | 0 | 1 | 0.8189 | 0.1811 |
| 9 | 10 | 15 | 0 | 8 | 0 | 1 | 0.8189 | 0.1811 |
| 10 | 11 | 9 | 0 | 4 | 0 | 1 | 0.8189 | 0.1811 |
| 11 | 12 | 5 | 0 | 4 | 0 | 1 | 0.8189 | 0.1811 |
| 12 | 13 | 1.5 | 0 | 3 | 0 | 1 | 0.8189 | 0.1811 |
| Interval (Lower; Upper) means the follow-up time; for example: lower=0; upper=1 means the interval between 0-1 year. | | | | | | | | |
| Effective sample size (n) means the total sample number in the CHM users. n = N-1/2 (NC). | | | | | | | | |
| Number of censored (NC) means the number of withdrawal or loss during the interval in the CHM users in this study. | | | | | | | | |
| Number of failed (NF) means the number of death during the interval in the CHM users in this study. | | | | | | | | |
| Conditional probability of failure (q) = NF/n. | | | | | | | | |
| Survival (p) = ㄇp = ㄇ(1-q). | | | | | | | | |
| Overall mortality = 1-Survival. | | | | | | | | |
